# Radiomics Using Fused PET and CT Data for Prognostic and Diagnostic Modeling in Head and Neck Cancer: A Systematic Review and Meta-Analysis

**DOI:** 10.3390/diagnostics16182900

**Published:** 2026-09-09

**Authors:** Abdulrahman Al Mopti, Abdulsalam Alqahtani, Ali H. D. Alshehri

**Affiliations:** 1 Department of Radiological Sciences, College of Applied Medical Sciences, Najran University, Najran 55461, Saudi Arabia; analmopti@nu.edu.sa (A.A.M.); aalqahtani@nu.edu.sa (A.A.); 2 Health Research Center, Najran University, Najran 55461, Saudi Arabia

**Keywords:** radiomics, 18F-FDG PET/CT, head and neck cancer, multimodal fusion, meta-analysis, prognostic modeling

## Abstract

**Objectives:** 18F-FDG PET/CT is central to head and neck cancer (HNC) care, yet most radiomic models use PET or CT alone. We quantified the discrimination of fused PET/CT models and what fusion adds. **Methods:** Following PRISMA 2020 and a registered protocol (PROSPERO CRD420261319855), seven sources were searched, supplemented by citation and proceedings searching. Reports sharing patients were grouped into independent cohort lineages, which served as the unit of analysis, and every synthesis included one analysis per lineage. The estimand was each report’s within-study difference between its fused model and its own comparator. Two confirmatory hypotheses were specified in the statistical analysis plan, with a Bonferroni threshold of 0.025. Random-effects models used REML with Hartung-Knapp; risk of bias used PROBAST+AI, reporting completeness METRICS v1.0 and diagnostic accuracy QUADAS-3. **Results:** 100 studies were included, arising from 31 independent cohort lineages. Fused models exceeded PET-only models by 0.042 (95% CI 0.014 to 0.070) on the concordance index (6 lineages; *p* = 0.0115), meeting the confirmatory threshold, and CT-only models by 0.037 (0.002 to 0.072; *p* = 0.0429), which did not. Pooled absolute discrimination was 0.711 (95% CI 0.659 to 0.763). Diagnostic evidence was reported by task and not pooled. Only 25 studies could contribute to any pooled estimate, chiefly because 71 of 100 fused estimates lacked a recoverable measure of precision; all were at high risk of bias in the analysis domain. **Conclusions:** Fusing PET and CT radiomic information improves survival discrimination in HNC, most securely against the PET-only baseline. The advantage over CT-only models was smaller and did not meet the multiplicity-adjusted criterion, and none was established for diagnostic classification. Incremental benefit depends on the comparator, the cohort and the endpoint; it does not follow from fusion itself.

## 1. Introduction

Head and neck cancer (HNC) ranks among the most common malignancies globally, with approximately 890,000 new cases and 450,000 deaths annually [1,2]. Clinical management relies on accurate staging, reliable prognostic stratification, and early identification of treatment-resistant disease. Although tumor, node, metastasis (TNM) staging remains the standard framework, it captures only limited biological heterogeneity that drives individual patient outcomes [2].

18F-fluorodeoxyglucose positron emission tomography/computed tomography (18F-FDG PET/CT) has become integral to HNC workup, informing initial staging, radiotherapy planning, treatment response assessment, and surveillance [3]. Beyond visual interpretation and conventional semi-quantitative parameters such as the maximum standardized uptake value (SUVmax) and metabolic tumor volume, radiomics offers the potential to extract high-dimensional features reflecting tumor phenotype at a level not accessible through standard clinical measures [4]. This approach rests on the hypothesis that spatial patterns of image intensity, encoded as texture, shape, and histogram features, carry biological information about tumor heterogeneity, aggressiveness, and microenvironment [5].

A growing body of literature has applied radiomics to PET/CT in HNC, targeting outcomes from overall survival to human papillomavirus (HPV) status prediction [6,7,8]. Most early work, however, extracted features from PET or CT independently, discarding the complementary information carried by the other modality. PET captures metabolic activity while CT provides structural detail; combining both should yield a more complete representation of tumor biology [7].

Several fusion strategies have been proposed. Image-level fusion combines PET and CT voxel data into a single composite image before feature extraction, for example through wavelet transforms or weighted summation [9,10]. Feature-level fusion extracts radiomic features independently from each modality and concatenates or jointly selects them for model training [11,12]. Decision-level fusion builds separate models on each modality and merges their predictions at the output stage, often through ensemble methods or evidential reasoning [13,14]. Each approach has theoretical strengths and limitations, and no consensus exists on which performs best.

A 2023 meta-analysis by Philip et al. [6] examined PET radiomics in HNC but focused on single-modality features without systematically evaluating CT fusion. To our knowledge, no review has specifically addressed whether fusing PET and CT radiomic data improves performance, nor systematically compared fusion strategies.

This review was designed to fill that gap. Our primary objective was to quantify what fusing PET and CT adds, as the within-study difference between each report’s fused model and its own single-modality comparator. Secondary objectives were to describe the absolute discrimination of fused models, compare fusion strategies, examine the influence of validation approach and study quality, and identify methodological gaps for future research.

## 2. Methods

This systematic review followed the Preferred Reporting Items for Systematic Reviews and Meta-Analyses (PRISMA) 2020 guidelines [15] and was registered prospectively in the International Prospective Register of Systematic Reviews (PROSPERO; CRD420261319855) on 16 February 2026.

### 2.1. Eligibility Criteria

Studies were eligible if they: (1) included HNC patients of any subsite imaged with both PET and CT; (2) developed or validated a radiomic model fusing PET and CT data at the image, feature, or decision level; (3) reported at least one discrimination metric (area under the receiver operating characteristic curve [AUC], concordance index [C-index], sensitivity, specificity, accuracy, or F1 score); (4) were original peer-reviewed English-language publications; and (5) for deep learning models, performed radiomics-equivalent analysis with explicit dual-modality fusion. Single-modality studies, those using only conventional PET parameters, or reporting only segmentation performance were excluded.

### 2.2. Information Sources and Search Strategy

PubMed/MEDLINE, the Cochrane Central Register of Controlled Trials (CENTRAL) through the Cochrane Library, the Applied Social Sciences Index and Abstracts (ASSIA) through ProQuest, the Web of Science Core Collection, IEEE Xplore, OpenAlex and Crossref were searched without date restriction. Embase and Scopus were named in the registered protocol and could not be searched: authenticated access attempts confirmed that neither platform is licensed to the institution, and no unofficial route was used. This amendment to the registered protocol is described in the Limitations. Searching was supplemented by backward and forward citation searching over every eligible report, an ISBN-anchored sweep of the complete proceedings of the HEad and neCK TumOR segmentation and outcome prediction (HECKTOR) challenge, and a corrections and retractions check. The search strategies are provided in full in the Supplementary Materials.

### 2.3. Selection Process

Records were deduplicated using a multi-pass algorithm (DOI matching, title-year matching, and fuzzy string similarity) implemented in Python 3 (Python Software Foundation, Wilmington, DE, USA). Two independent reviewers screened titles and abstracts; records advanced when either reviewer judged them potentially eligible. Full texts were sought for 192 records and retrieved for 189; 3 could not be obtained despite multiple attempts and were excluded as unretrievable (E11). Full-text screening was performed independently by both reviewers for every retrieved report, and disagreements were resolved by protocol-based adjudication against the source document.

### 2.4. Data Extraction

Analysis-bearing data were extracted independently by two reviewers into a pre-specified form covering study characteristics, population, imaging parameters, the radiomics pipeline, model performance for fused and single-modality models, and validation approach. Every disagreeing field was settled by returning to the primary report, and each comparator was recorded with a page-and-table locator and classified by how closely it matches the fused arm.

### 2.5. Risk of Bias Assessment

Risk of bias was assessed using the Prediction model Risk Of Bias ASsessment Tool for artificial intelligence (PROBAST+AI) [16] for all 100 appraised studies, reporting completeness using the METhodological RadiomICs Score (METRICS) v1.0 [17] applied item by item, and diagnostic accuracy using the Quality Assessment of Diagnostic Accuracy Studies tool, version 3 (QUADAS-3) [18] for the 12 diagnostic estimates. All three are applied on positive evidence only: a domain is rated low, or an item answered yes, solely where the report supplies the necessary evidence. No overall numerical quality score is computed, because none of the three instruments defines one. Full assessments are in the Supplementary Materials.

### 2.6. Data Synthesis and Statistical Analysis

Random-effects meta-analyses used restricted maximum likelihood (REML) estimation with the Hartung-Knapp adjustment, implemented in the meta (version 8.2.1) [19] and metafor (version 4.8.0) [20] packages in R (version 4.3.3R Foundation for Statistical Computing, Vienna, Austria). Reports drawing on the same patients are not independent replications: cohort provenance was mapped from each report’s own statement of its data source, reports were grouped into independent cohort lineages keyed to the cohort on which the estimate is evaluated, and every synthesis admits one analysis per lineage. The representative is selected by the largest independently held-out evaluation set, then the earliest publication year, then the record identifier; the rule never consults the size or direction of the effect. Estimates were pooled separately by metric and by clinical question. The estimand, fixed in the statistical analysis plan, was the within-study difference between a report’s fused model and its own comparator. Where a study reported several fused models, endpoints, time points or validation cohorts, the model the authors designated as primary was extracted, preferring external validation, then an independent test set, then cross-validation. The best-performing result across experiments was not selected. Standard errors were taken as reported, derived from a reported interval of stated level, or, for the area under the curve only, derived by the method of Hanley and McNeil [21]; that method was never applied to a concordance index and was refused where the unit of evaluation was the node, lesion or region of interest. No standard error was imputed for any study. The two arms of each difference are measured on the same patients and are correlated; because that correlation is never reported, the variance of each difference was evaluated across a grid of assumed correlations (0, 0.25, 0.50, 0.75, 0.90) with 0.50 as the reference. Each concordance-index difference is also expressed as Somers’ D (D = 2C − 1), an exact linear re-expression of the concordance scale. Two confirmatory prognostic comparisons, fused versus CT-only and fused versus PET-only on the concordance index, were specified in the statistical analysis plan, which was finalized and dated before data extraction for the enlarged evidence base began (Supplementary Table S8); they were tested against a Bonferroni threshold of 0.025. Every other estimate is reported for estimation, and no *p* value from it is compared to any threshold.

Heterogeneity was quantified using I-squared with its confidence interval (CI), Cochran Q, tau-squared, and prediction intervals where five or more lineages contribute. Subgroup analyses, specified in the analysis plan, examined fusion strategy, validation approach, endpoint family, cancer subsite, and feature extraction method, each computed on the primary one-analysis-per-lineage set and each requiring three independent lineages. Meta-regression and tests for small-study effects require at least ten independent contributions; no analysis reaches that number once cohort dependence is respected, so neither was performed. Sensitivity analyses omitted each lineage in turn, restricted to externally or independently validated analyses, restricted to analyses resting on a reported standard error, and entered every contributing analysis without modeling dependence.

Claude Opus 5 (Anthropic, San Francisco, CA, USA) was used to edit and revise the language of the manuscript. It was not used for study selection, data extraction, risk-of-bias appraisal or the statistical analysis, and every edit was reviewed and approved by the authors.

## 3. Results

### 3.1. Study Selection

**Figure 1:**
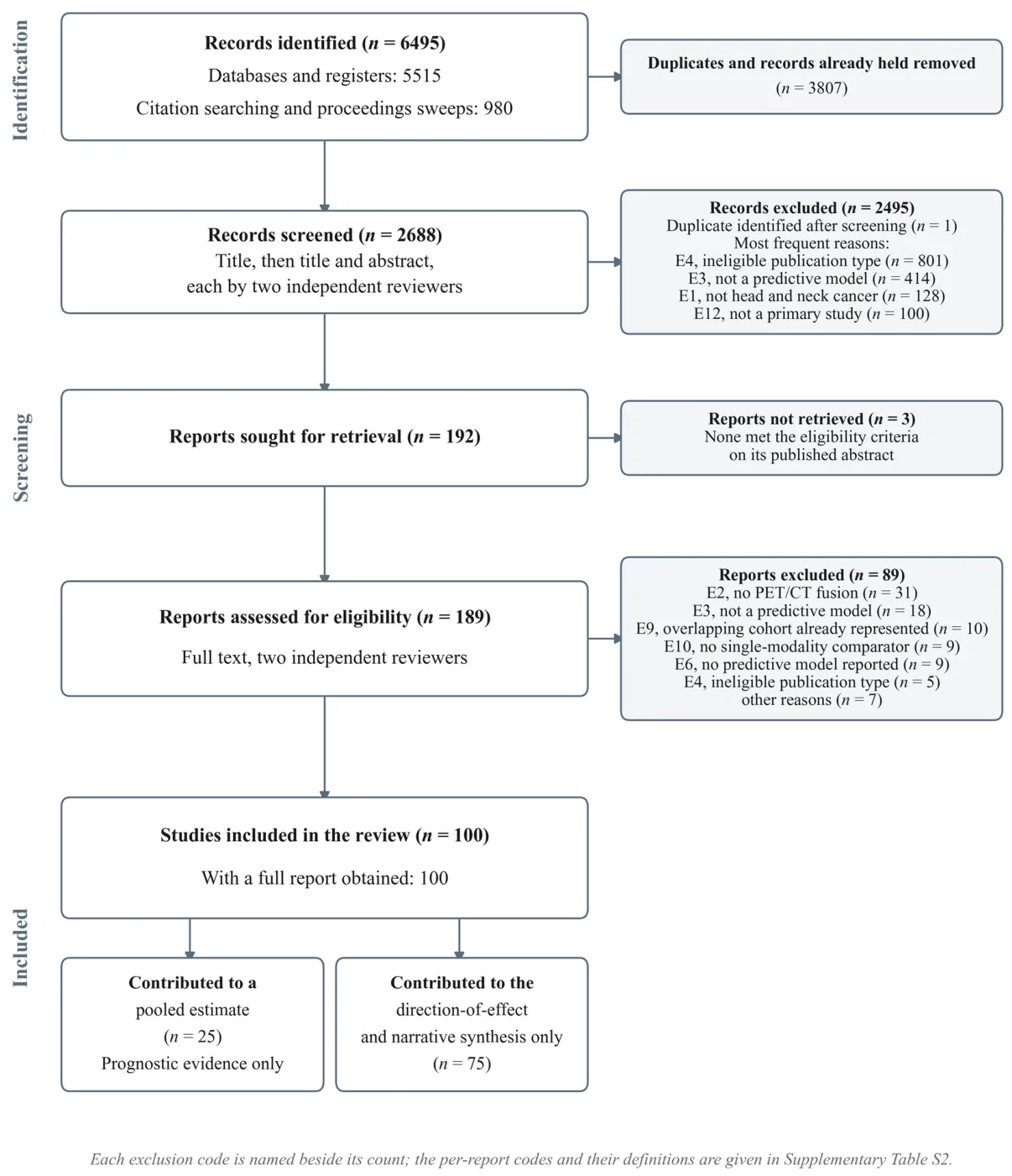
PRISMA 2020 flow diagram showing study identification, screening, and inclusion.

The searches identified 5515 records from databases and 980 from citation searching and proceedings sweeps. After removing 3807 duplicates and records already held, 2688 were screened, of which 2495 were excluded and 1 was identified as a duplicate of an already-screened record. Of 192 reports sought for retrieval, 3 could not be accessed (E11), leaving 189 full texts assessed for eligibility. Of these, 89 were excluded, leaving 100 studies that met the eligibility criteria [7,8,9,10,11,12,13,14,22,23,24,25,26,27,28,29,30,31,32,33,34,35,36,37,38,39,40,41,42,43,44,45,46,47,48,49,50,51,52,53,54,55,56,57,58,59,60,61,62,63,64,65,66,67,68,69,70,71,72,73,74,75,76,77,78,79,80,81,82,83,84,85,86,87,88,89,90,91,92,93,94,95,96,97,98,99,100,101,102,103,104,105,106,107,108,109,110,111,112,113] (Figure 1).

### 3.2. Characteristics of Included Studies

The 100 studies were published between 2009 and 2026. Cohorts ranged from 50 to 889 patients, with a median of 325. A single patient total is not reported because reports sharing a cohort would inflate it. Studies whose unit of analysis is the lymph node, lesion or region of interest are identified as such, and their estimates are never read beside per-patient estimates.

Of the 100 studies with an adjudicated extraction, 88 addressed a prognostic question and 12 a diagnostic one. Characteristics are given per study in the data workbook (sheet S1_characteristics). Two features of this evidence base govern how much of it can be synthesized, and Supplementary Figure S6 shows both. The first is cohort dependence: the 100 extractable studies arise from 31 independent cohort lineages, and one lineage, the public collections distributed through The Cancer Imaging Archive and the HECKTOR challenge, accounts for 66 of them. A count of reports would therefore overstate the independent evidence severalfold, because one cohort can supply many reports.

The second feature is the reporting of precision. Of 100 fused estimates, 26 were accompanied by a usable standard error or confidence interval and 3 permitted one to be validly derived, leaving 71 reported as a point estimate with no recoverable measure of uncertainty. In consequence, 25 studies contribute to a pooled estimate (Supplementary Figure S6B). Every pooled figure below is an availability sample, is reported with the number of lineages it rests on, and does not describe the literature as a whole.

### 3.3. Fusion Strategies

Fusion was implemented at the level of features in 48 studies, of images in 25, across multiple levels in 21 and of model outputs in 6. Each was recorded from the primary report and is given per study in the data workbook.

### 3.4. Validation Approaches

Validation settings are given per study in the data workbook and summarized in Table 1. Every analysis contributing to a pooled difference was evaluated at external validation or on an independent test set; no cross-validated estimate enters a difference pool.

### 3.5. Pooled Performance of Fused PET/CT Models

Pooled absolute discrimination of fused prognostic models was 0.711 (95% CI 0.659 to 0.763) for the concordance index across 9 independent lineages (16 contributing analyses; I-squared 86%, 95% CI, 76% to 92%; 95% prediction interval, 0.559 to 0.862) and 0.813 (95% CI, 0.733 to 0.893) for the area under the curve across 7 lineages (9 contributing analyses; I-squared 57%, 95% CI, 0% to 81%; 95% prediction interval, 0.644 to 0.982); both are shown study by study in Supplementary Figure S7. These describe how fused models performed. They carry no information about what fusion contributed, and the width of the prediction intervals shows how little a single figure constrains what a new cohort would yield. The 12 diagnostic estimates are reported individually by clinical task in the data workbook and are not pooled: the target conditions are different clinical questions with different prevalences, and no task reaches the registered minimum of three sufficiently independent analyses at a common unit of analysis.

### 3.6. Fusion Benefit over Single-Modality Models

**Figure 2:**
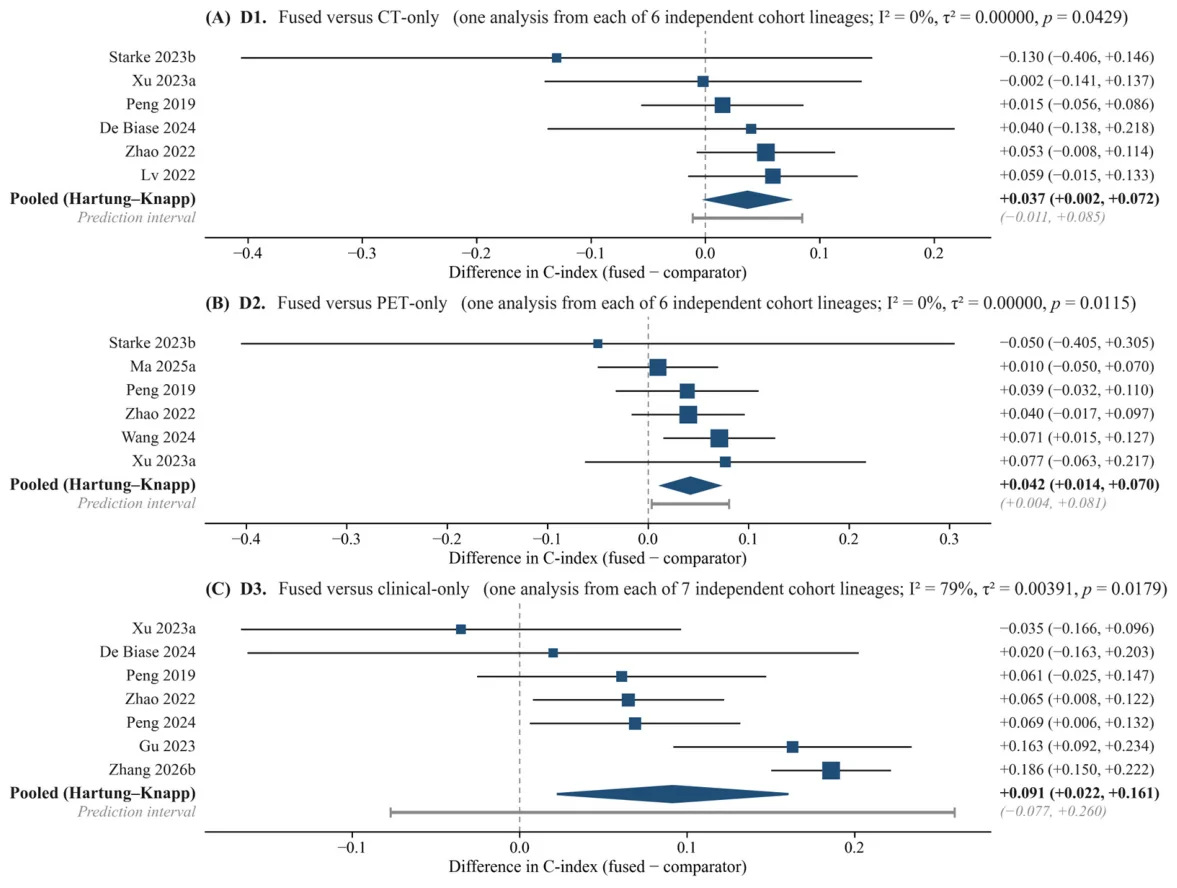
Forest plot of the primary endpoint: the within-study difference in concordance index (C-index) between each study’s fused model and its own comparator, against CT-only (panel (**A**), analysis D1), PET-only (panel (**B**), D2) and clinical-only (panel (**C**), D3) models, one analysis from each independent cohort lineage. Squares mark each study’s difference, with area proportional to its inverse-variance weight; horizontal lines are 95% confidence intervals; the diamond is the pooled random-effects estimate with its Hartung–Knapp confidence interval; the grey bar beneath it is the 95% prediction interval; the dashed vertical line marks no difference. Each panel header gives the number of contributing lineages, I^2^, τ^2^ and the *p* value of the pooled difference. Studies shown: Peng 2019 [12]; De Biase 2024 [34]; Gu 2023 [43]; Lv 2022 [60]; Ma 2025a [67]; Peng 2024 [78]; Starke 2023b [89]; Wang 2024 [96]; Xu 2023a [102]; Zhang 2026b [109]; Zhao 2022 [110]. CT, computed tomography; PET, positron emission tomography.

Paired within-study analyses were performed separately for each metric, on one analysis per independent cohort lineage, and a Bonferroni threshold of 0.025 was applied across the confirmatory family of two specified in the analysis plan (Supplementary Table S8). Fused models exceeded PET-only models by 0.042 (95% CI 0.014 to 0.070) on the concordance index across 6 lineages (10 contributing analyses; multiplicity-compatible, 97.5% CI, 0.0079 to 0.0765; *p* = 0.0115), meeting the confirmatory threshold; the 95% prediction interval, 0.004 to 0.081, excludes zero (Table 1, Figure 2). The difference from CT-only models was 0.037 (95% CI, 0.002 to 0.072) across 6 lineages (10 contributing analyses; 97.5% CI, −0.0064 to 0.0800; *p* = 0.0429), which does not meet the threshold, and its prediction interval, −0.011 to 0.085, includes zero. Fused models exceeded models built from clinical variables alone by 0.091 (95% CI, 0.022 to 0.161) across 7 lineages, with the highest heterogeneity of any analysis here (I-squared 79%, 95% CI, 57% to 90%). Expressed as a standardized concordance-scale effect, Somers’ D, the advantage over PET-only models was 0.084 and over CT-only models was 0.074. On the area under the curve, four paired comparisons were available for each comparator; the analysis plan permits an exploratory contrast on that metric only at five or more commensurate pairs, so each is reported individually in the data workbook.

The asymmetry is informative, and it runs opposite to the direction usually assumed. The advantage over PET-only models is the one that meets the confirmatory criterion, while the advantage over CT-only models does not. One reading is that PET carries much of the prognostic signal in this disease, so that a fused model must improve on an already informative baseline, and the fact that it measurably does is the more demanding test. The CT comparison starts from a weaker baseline, where a larger apparent gain is easier to obtain and harder to separate from differences in how the CT arm was built. The intervals are compatible with that reading without establishing it.

### 3.7. Subgroup Analyses

Prognostic studies include those reporting a concordance index pooled at 0.711 (9 lineages) and those reporting an area under the curve at 0.813 (7 lineages). These strata are not compared with one another, because an area under the curve and a concordance index are different measures and a between-group test across them would not be interpretable. Diagnostic estimates are not pooled at any level.

Subgroup estimates by fusion strategy, feature type, validation setting, endpoint family and tumor subsite are shown in Supplementary Figure S5, with the full grids available from the corresponding author upon reasonable request. Each was computed on the primary one-analysis-per-lineage set, so a level had to reach three independent lineages to appear, and each is reported as an estimate; no *p* value from a subgroup is compared to any threshold. Across all five pools, only two axes returned more than one qualifying level, both within absolute prognostic discrimination, so in most cases no comparison between levels was available at all. Where two levels did qualify, comparing deep-learning with handcrafted features and external with independent-test validation, their intervals overlapped. A single endpoint family qualified in two analyses, locoregional control in the absolute AUC pool (five lineages) and progression-free survival in the clinical-comparator pool (three lineages); neither analysis offered a second family to compare it with.

Meta-regression was not performed. The protocol requires ten independent contributions before a moderator analysis is interpretable, and no analysis in this review reaches that number once cohort dependence is respected.

### 3.8. Risk of Bias

**Figure 3:**
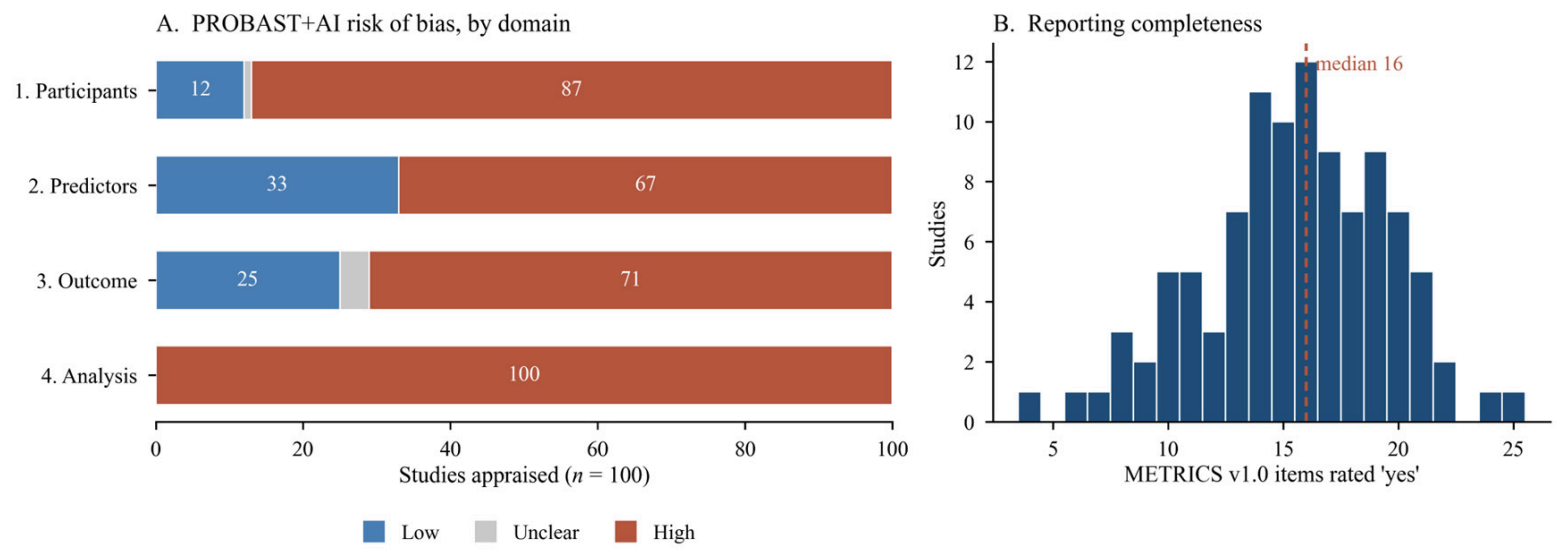
Quality assessment of included studies. (**A**) PROBAST+AI risk of bias by domain. (**B**) Reporting completeness by METRICS v1.0.

PROBAST+AI rated all 100 appraised studies at high overall risk of bias, in every case through the analysis domain, which was rated high without exception (Figure 3). The participants domain was rated low in 12 studies, the predictors domain in 33 and the outcome domain in 25. The recurring reasons for the analysis rating were the absence of correction for the optimism induced by selecting among many candidate models, the absence of calibration assessment, and the absence of the event counts needed to judge whether the outcome events could support the number of candidate predictors. The consistency of this finding removes the usual mitigation of restricting synthesis to low-risk studies, because no low-risk subgroup exists.

Reporting completeness assessed by METRICS v1.0 varied widely: of the thirty items, studies answered between 4 and 25 affirmatively (median 16). No total score is computed, because the instrument does not define one, and conditionality is enforced in both directions, so an item which cannot apply to a design is recorded as not applicable.

For the diagnostic estimates, QUADAS-3 was applied to all 12 and is reported per study in Supplementary Table S4. Prognostic model studies are outside the instrument’s scope and are not assessed with it.

Certainty of the evidence was considered for each outcome against risk of bias, inconsistency, indirectness, imprecision and reporting bias. Because every appraised study is at high risk of bias through the analysis domain, no outcome is judged above moderate certainty, and the absolute discrimination pools, which additionally carry substantial heterogeneity and do not address incremental value, are judged very low.

### 3.9. Sensitivity Analyses

The advantage over PET-only models was positive under every sensitivity analysis specified in the analysis plan. Omitting each lineage in turn moved the estimate between 0.031 and 0.052, and the confidence interval excluded zero in every one of these fits. Across the full grid of assumed correlations, the largest *p* value was 0.016, inside the confirmatory threshold throughout. The comparison against CT-only models was less stable: leave-one-out moved it between 0.028 and 0.045, and at the most adverse correlation assumed the *p* value rose to 0.077. Supplementary Figure S8 shows the whole correlation grid.

Two restrictions removed no analysis from any difference pool: every contributing analysis was evaluated at external validation or on an independent test set, and every standard error entering these pools was either reported directly or recovered algebraically from a printed confidence interval, none resting on an approximation from class frequencies. Entering every contributing analysis instead of one per lineage, which does not model the dependence between reports sharing a cohort, gives a larger CT-only difference (0.043) and a similar PET-only difference (0.040). The gap between the two fits for the CT comparison indicates how much of its apparent support rests on counting shared cohorts more than once.

### 3.10. Publication Bias

Small-study effects could not be assessed. The protocol requires ten independent contributions before a funnel plot or a test of asymmetry is interpretable, and no analysis in this review reaches that number once cohort dependence is respected. Publication bias in the literature where nearly every report favors the studied intervention is likely and can be neither quantified nor excluded.

## 4. Discussion

This systematic review and meta-analysis provide the first comprehensive quantitative assessment of whether fusing PET and CT radiomic data improves predictive modeling in HNC. Across 100 studies, whose 100 extractable reports arise from 31 independent cohort lineages, fused models improved discrimination over a PET-only baseline by 0.042 on the concordance index, meeting the confirmatory criterion. The advantage over CT-only models, 0.037, is positive but does not meet that criterion and is sensitive to the assumed correlation between the paired arms.

### 4.1. The Case for Fusion

Fusion adds measurable discrimination to survival models, and the increment is small. Among analyses reporting a concordance index, fused models exceeded PET-only models by 0.042 and CT-only models by 0.037, differences in the region of three to four points of concordance. Only the PET-only margin survives correction for multiplicity. Neither is large enough to change practice on its own: an improvement of that size shifts few individual patients across a decision threshold, and the prediction intervals are wide enough to include increments too small to matter clinically.

Benefit asymmetry is itself an important finding, and it inverts the emphasis usually placed on adding metabolic information to anatomy. That the PET comparison is the more secure of the two suggests that PET features carry much of the prognostic signal in this disease, so that a fused model must improve on an already informative baseline. The CT comparison starts from a weaker baseline, where a larger apparent gain is easier to obtain and harder to separate from differences in how the CT arm was built.

### 4.2. Comparison with Prior Reviews

Philip et al. [6] reviewed 31 PET radiomics studies in HNC and found that combined clinical–PET models achieved acceptable discrimination, but their analysis was limited to PET-derived features and did not evaluate CT fusion. Our review extends this work by specifically quantifying the incremental value of adding CT radiomics to PET-based models. In head and neck cancer, Lv et al. [7] and Salmanpour et al. [9,10] demonstrated that image-level and tensor-based PET/CT fusion outperforms single-modality approaches for survival prediction. A 2026 review of AI models for lung cancer staging found integrated PET/CT models superior, particularly for nodal staging [114]. Our HNC findings are consistent with this broader cross-tumor pattern, suggesting the fusion benefit may be generalizable.

A recent review of multimodal radiomics across cancers concluded fusion better captures disease biology and generalizes more robustly [115]. Our quantitative pooling provides numerical support for this observation in HNC.

### 4.3. Fusion Strategy Comparison

Feature-level fusion dominated (48 studies), likely due to implementation simplicity. Image-level fusion (25) was associated with several top-performing models, particularly through deep learning with cross-attention or transformers. Decision-level fusion (6) showed no clear performance advantage. No subgroup comparison between strategies reached the three independent lineages the protocol requires, so no strategy can be recommended over another on this evidence.

The comparison by Lv et al. [7], who systematically evaluated image-level, matrix-level and feature-level fusion within a single multicentre cohort, remains one of the most informative studies in this area. They found wavelet-based image fusion optimal for metastasis-free survival, though the advantage over feature-level fusion was small. No subgroup comparison in the present review reached the three independent lineages the protocol requires, so that single study remains the best available evidence on which fusion level to prefer, and it is one cohort.

### 4.4. Methodological Quality

Risk of bias was high in all 100 appraised studies, and it was concentrated. Participant selection, predictors and outcome definition were rated low risk in a substantial minority of studies; the entire burden falls on the analysis domain, which was rated high without exception. The problem in this literature is not how patients were chosen or how outcomes were defined, but how models were built and evaluated.

Because every study is at high risk of bias through the analysis domain, no low-risk subgroup exists to restrict to, and the usual mitigation is unavailable. The estimates here should be read as summarizing a literature that is optimistically biased to an extent that cannot be quantified from what is published. The most consequential finding of this review is not an effect size: it is that 71 of 100 studies reported the performance of their fused model without a recoverable measure of uncertainty, so that only 25 of 100 could contribute to a pooled estimate.

### 4.5. Limitations

Several limitations should be acknowledged. Although direct Embase and Scopus searching remained unavailable after documented access attempts, complementary indexing and citation-search approaches were used to reduce the associated coverage risk; the possibility of missed reports cannot be excluded. Small-study effects could not be assessed, because no analysis reaches the ten independent contributions the protocol requires. The pooled differences rest on between 6 and 7 independent lineages. The correlation between the paired arms is not reported by any study, and although the full grid of assumptions is examined, the CT-only comparison is sensitive to it. Three properties of the model deserve plain statement: the Hartung-Knapp interval is a small-sample correction whose calibration is not guaranteed at six lineages with a heterogeneity estimate on its boundary; pooling on the raw scale of a bounded index assumes a normal working model for a quantity that cannot exceed one; and an I-squared of zero at six lineages means the estimate reached its boundary, and the interval reported beside it shows how little these data constrain heterogeneity.

### 4.6. Implications for Practice and Future Research

The evidence assembled here supports the integration of both PET and CT radiomic features in predictive models for HNC. Practically, this means that researchers developing new radiomics models from PET/CT data should extract features from both modalities; defaulting to a single modality forgoes information the examination already contains. Extracting features from both modalities adds little to a pipeline that already processes the study, since both series are acquired in the same examination.

The distribution of practice across these 100 studies indicates where effort would be best spent, and permits recommendations more specific than a call for better methods. Report a measure of uncertainty with every performance estimate; an interval on a concordance index costs nothing and is what makes a result usable. Report the single-modality models against which the fused model is claimed to improve, evaluated on the same patients at the same stage. State the provenance of the cohort precisely enough that a reader can tell whether it overlaps another published series. Constrain dimensionality before modeling, and report calibration, which determines whether a model’s outputs can be acted upon and is almost never given.

## 5. Conclusions

Across 100 studies drawn from 31 independent cohort lineages, fused PET/CT radiomic models achieved moderate discrimination for survival prediction (pooled concordance index 0.711, 95% CI 0.659 to 0.763). Fusion improved on a PET-only baseline by 0.042, a margin that met the multiplicity-adjusted confirmatory criterion and remained positive under every sensitivity analysis. The improvement over CT-only radiomics was smaller and did not meet that criterion, while comparisons with clinical models were heterogeneous. Diagnostic evidence remained too sparse and cohort-dependent for quantitative synthesis. These findings support the potential value of multimodal radiomics while emphasizing the need for independent validation, transparent uncertainty reporting and methodologically rigorous comparative models.

## Figures and Tables

**Table 1:** Results of the principal analyses.

ID	Analysis	Independent Cohorts	Contributing Analyses	Estimate (95% CI)	Somers’ D (95% CI)	*p*	I-Squared % (95% CI)	Tau-Squared	95% Prediction Interval
A1	Absolute fused prognostic C-index	9	16	0.711 (0.659 to 0.763)	—	—	86 (76 to 92)	0.00372	0.559 to 0.862
A2	Absolute fused prognostic AUC	7	9	0.813 (0.733 to 0.893)	—	—	57 (0 to 81)	0.00379	0.644 to 0.982
D1	Delta C-index, fused vs. CT-only	6	10	0.037 (0.002 to 0.072)	0.074 (0.003 to 0.144)	0.0429	0 (0 to 75)	0.00000	−0.011 to 0.085
D2	Delta C-index, fused vs. PET-only	6	10	0.042 (0.014 to 0.070)	0.084 (0.029 to 0.140)	0.0115	0 (0 to 75)	0.00000	0.004 to 0.081
D3	Delta C-index, fused vs. clinical-only	7	8	0.091 (0.022 to 0.161)	0.183 (0.044 to 0.321)	0.0179	79 (57 to 90)	0.00391	−0.077 to 0.260
D4	Delta AUC, fused vs. CT-only	4	4	Not pooled ‡	—	—	—	—	—
D5	Delta AUC, fused vs. PET-only	4	4	Not pooled ‡	—	—	—	—	—
D6	Delta AUC, fused vs. clinical-only	4	4	Not pooled ‡	—	—	—	—	—

Every estimate is fitted to one analysis per independent cohort lineage, which is the primary unit of this review; the number of contributing analyses is given beside it and is not a count of independent contributions. All fits are random-effects with tau-squared by restricted maximum likelihood and a Hartung-Knapp confidence interval, on the raw scale of the discrimination index. Somers’ D is an exact linear re-expression of a difference in the concordance index, D = 2C − 1, and is not a standardized mean difference. The confirmatory family comprises exactly two hypotheses, D1 and D2, tested at a Bonferroni alpha of 0.025; every other estimate is reported for estimation and its *p* value, where shown, is not compared to any threshold. Absolute analyses carry no *p* value, because the protocol specifies no null hypothesis for absolute discrimination. ‡ The three area-under-the-curve difference analyses are not pooled: the analysis plan permits an exploratory contrast on that metric only at five or more commensurate pairs, and four are available. They are reported per cohort in the data workbook.

## Data Availability

The data presented in this study are available from the corresponding author upon reasonable request.

## Supplementary Materials

The following supporting information can be downloaded at: https://www.mdpi.com/article/10.3390/diagnostics16182900/s1, Table S1: Information sources and records retrieved, by source; Table S2: The review corpus: every included study and every report removed at final revalidation; Table S3: PROBAST+AI risk of bias, all 100 appraised studies; Table S4: QUADAS-3 assessment, 12 diagnostic estimates; Table S5: Sensitivity analyses; Table S6: METRICS v1.0 reporting completeness; Table S7: The completed PRISMA 2020 checklist; Table S8: Deviations from the registered protocol; Table S9: Guide to the supplementary data workbook, followed by the verbatim search strategies, source by source; Figure S1: The included studies described: (A) publication year, (B) cohort size on a logarithmic scale with the median marked, (C) how the two modalities were combined; Figure S2: PROBAST+AI risk of bias, domain by domain, for every appraised study; Figure S3: QUADAS-3 for the diagnostic estimates; Figure S4: Leave-one-out for every pooled analysis, one row per omitted cohort lineage; Figure S5: Every subgroup estimate reaching three independent cohort lineages, computed on the primary one-analysis-per-lineage set; Figure S6: Why the included reports are not that many independent pieces of evidence: (A) reports drawn from each independent cohort lineage, on a logarithmic scale, (B) how many studies could enter a pooled estimate, and where the rest were lost; Figure S7: Absolute discrimination of the fused model, concordance index (A1) and area under the curve (A2), one analysis from each independent cohort lineage; Figure S8: Each difference across the grid of assumed correlations between the paired arms, with the heterogeneity that accompanies each assumption; Supplementary Data Workbook (Excel): twelve sheets carrying every per-study row behind the review (S0_corpus, S1_characteristics, S2_performance_and_comparators, S3_direction_of_effect, S4_pool_membership, S5_PROBAST_AI, S6_METRICS_v1, S7_QUADAS_3, S8_sensitivity, S9_leave_one_out, S10_derived_standard_errors, S11_PRISMA_by_wave).
