# Supplementary material for: Radiomics Using Fused PET and CT Data for Prognostic and Diagnostic Modeling in Head and Neck Cancer: A Systematic Review and Meta-Analysis
Source: Diagnostics (Basel). 2026 Sep 9;16(18):2900. doi: 10.3390/diagnostics16182900 (PMC13605430; doi:10.3390/diagnostics16182900)
Supplement: Supplementary file 1 [file diagnostics-16-02900-s001.zip › Supplementary_Materials.pdf]

Supplementary Materials

Radiomics Using Fused PET and CT Data for Prognostic and Diagnostic Modeling in Head and Neck Cancer: A Systematic Review and Meta-analysis

PROSPERO CRD420261319855

Contents

Table S1. Information sources and records retrieved, by source.

Table S2. The review corpus: every included study and every report removed at final revalidation.

Table S3. PROBAST+AI risk of bias, all 100 appraised studies.

Table S4. QUADAS-3 assessment, 12 diagnostic estimates.

Table S5. Sensitivity analyses.

Table S6. METRICS v1.0 reporting completeness.

Figure S1. The included studies described: (A) publication year, (B) cohort size on a logarithmic scale with the median marked, (C) how the two modalities were combined.

Figure S2. PROBAST+AI risk of bias, domain by domain, for every appraised study. Negative-evidence scoring: a domain is rated low only where the report supplies the evidence.

Figure S3. QUADAS-3 for the diagnostic estimates. Prognostic model studies are outside the instrument's scope and are not assessed with it.

Figure S4. Leave-one-out for every pooled analysis, one row per omitted cohort lineage. The shaded band is the full-set estimate with its confidence interval.

Figure S5. Every subgroup estimate reaching three independent cohort lineages, computed on the primary one-analysis-per-lineage set and reported as an estimate; no between-level test is performed.

Figure S6. Why the included reports are not that many independent pieces of evidence. (A) Reports drawn from each independent cohort lineage, on a logarithmic scale. (B) How many studies could enter a pooled estimate, and where the rest were lost.

Figure S7. Absolute discrimination of the fused model, concordance index (A1) and area under the curve (A2), one analysis from each independent cohort lineage.

Figure S8. Each difference across the grid of assumed correlations between the paired arms, with the heterogeneity that accompanies each assumption.

Table S7. The completed PRISMA 2020 checklist.

Table S8. Deviations from the registered protocol.

Table S9. Guide to the supplementary data workbook. The verbatim search strategies follow it, source by source.

Table S1. Information sources and records retrieved, by source.

A source is described as searched only where a search was run; where access was refused, the attempt and the platform's own wording are recorded instead. The full Boolean strategy is reproduced verbatim after Table S9 for every source whose executed string was retained. Sources searched programmatically are recorded with their query parameters below, and one platform search whose exact string was not retained is documented with its interface, field settings and yield.

Source	Interface	Date searched	Retrieved	Contribution
PubMed/MEDLINE	NCBI E-utilities and the web interface; no filters, no date limit	30 July 2026; updated 17 August 2026	1,232 (delta 6)	searched; full strategy reproduced below
MEDLINE (Ovid syntax)	Recorded for reproducibility; MEDLINE was searched through PubMed	30 July 2026	see PubMed	searched; full strategy reproduced below
Cochrane CENTRAL	Cochrane Library Advanced Search	registered wave	88	searched; full strategy reproduced below
Cochrane Library	Cochrane Library, all databases	registered wave	included in 88	searched; full strategy reproduced below
ASSIA	ProQuest, Saudi Digital Library	registered wave	101	searched; full strategy reproduced below
Web of Science Core Collection	Advanced Search Query Builder; Editions: All; no date range, no language or document-type limit	30 July 2026, re-run 17 August 2026	664	searched; full strategy reproduced below
IEEE Xplore	Command Search, field "All Metadata" throughout; no date range, no document-type filter, Show: All Results, not Subscribed Content	17 August 2026	80	The query was three blocks joined by AND, translated from the strategy of record into IEEE command syntax within the platform's limit of 25 terms per clause: 14 population terms, 10 modality terms, 17 method terms. The exact string as pasted was not saved to the project record; it is therefore not reproduced here, and it has not been reconstructed. The 80 retrieved records, their DOIs and the full screening decisions are in the review data, so the yield is auditable even though the string is not.
OpenAlex	API, programmatic query over title, abstract and concept fields	17 August 2026	largest single contributor to the update wave	Executed programmatically, not pasted into a search box; the query is embodied in the script filed with the review data.
Crossref	API; publisher-member sweep, all-publisher sweep, and an ISBN-anchored sweep of the complete HECKTOR proceedings	31 July 2026; proceedings 17 August 2026	2,400 swept; 65 chapters	Executed programmatically, not pasted into a search box; the queries are embodied in the scripts filed with the review data.
Embase	embase.com/search/quick, signed in to an authenticated Elsevier account	17 August 2026	not searched	Entitlement absent. The platform returned: "You are successfully signed in to Elsevier. Your account doesn't have direct access to Embase, but you might have access to Embase via your institution." No search interface was reachable, so no search was run and none is claimed.
Scopus	scopus.com advanced search form, same authenticated Elsevier account	17 August 2026	not searched	Entitlement absent. The advanced search form does not load for an unentitled account; the request redirects to Scopus Preview, which exposes source and author records only and cannot run a document search.

Table S2. The review corpus: every included study and every report removed at final revalidation.

Each row carries its status, its denominators, and, where excluded, its registered code and reason. The 89 reports excluded at full-text assessment are itemised by wave and registered code in workbook sheet S11_PRISMA_by_wave, and the totals are asserted against Figure 1. Exclusion codes follow the registered protocol: E1 not head and neck cancer; E2 no PET/CT fusion; E3 not a predictive model; E4 ineligible publication type; E5 no eligible outcome; E6 no predictive model reported; E7 no discrimination metric; E8 segmentation only; E9 overlapping cohort already represented; E10 no single-modality comparator; E11 full text not retrievable; E12 not a primary study. No study appears in a denominator it did not contribute to.

**record_id**	**Ref. no.**	**Study**	**Status**	**Extracted**	**Appraised**	**In a pooled analysis**	**Note**
record_id	Ref. no.	Study	Status	Extracted	Appraised	In a pooled analysis	Note
D00006	11	Lin 2026a	included	yes	yes	yes	
D00010	108	Zhang 2026a	included	yes	yes	no	
D00057	67	Ma 2025a	included	yes	yes	yes	
D00058	68	Ma 2025b	included	yes	yes	yes	
D00073	99	Wu 2024a	included	yes	yes	no	
D00084	76	Oka 2024	included	yes	yes	no	
D00102	100	Wu 2024b	included	yes	yes	no	
D00109	57	Li 2024	included	yes	yes	yes	
D00120	34	De Biase 2024	included	yes	yes	yes	
D00126	98	Wu 2023	included	yes	yes	no	
D00133	9	Salmanpour 2023a	included	yes	yes	no	
D00156	61	Lv 2023	included	yes	yes	no	
D00159	94	Wang 2023a	included	yes	yes	yes	
D00162	54	Kulanthaivelu 2022	included	yes	yes	no	
D00163	56	Le 2022	included	yes	yes	no	
D00165	14	Zhang 2022	included	yes	yes	yes	
D00169	42	Gu 2022	included	yes	yes	yes	
D00176	90	Wang 2022a	included	yes	yes	yes	
D00178	71	Meng 2022a	included	yes	yes	no	
D00189	75	Naser 2022	included	yes	yes	no	
D00227	30	Chen 2021	included	yes	yes	no	
D00246	47	Haider 2021	included	yes	yes	no	
D00249	111	Zhong 2021	included	yes	yes	yes	
D00256	13	Wang 2020	included	yes	yes	yes	
D00276	7	Lv 2020	included	yes	yes	no	
D00303	12	Peng 2019	included	yes	yes	yes	
D00310	29	Chen 2019	included	yes	yes	no	
D00332	32	Crispin-Ortuzar 2018	included	yes	yes	no	
D00338	25	Bogowicz 2017	included	yes	yes	no	
D00378	106	Yu 2009	included	yes	yes	no	

D00459	97	Wang 2025	included	yes	yes	no	
D00462	104	Yang 2025	included	yes	yes	no	
D00479	96	Wang 2024	included	yes	yes	yes	
D00481	82	Saikia 2024	included	yes	yes	no	
D00485	95	Wang 2023b	included	yes	yes	yes	
D00487	43	Gu 2023	included	yes	yes	yes	
D00488	102	Xu 2023a	included	yes	yes	yes	
D00489	50	Huynh 2023	included	yes	yes	no	
D00494	103	Xu 2023b	included	yes	yes	no	
D00497	35	Ding 2023	included	yes	yes	yes	
D00498	88	Starke 2023a	included	yes	yes	yes	
D00505	10	Salmanpour 2023b	included	yes	yes	no	
D00507	83	Salahuddin 2023	included	yes	yes	no	
D00509	89	Starke 2023b	included	yes	yes	yes	
D00514	110	Zhao 2022	included	yes	yes	yes	
D00521	27	Brancati 2022	included	yes	yes	no	
D00523	60	Lv 2022	included	yes	yes	yes	
D00534	45	Haider 2020a	included	yes	yes	no	
D00539	46	Haider 2020b	included	yes	yes	no	
D00546	62	Lv Wenbing 2019	included	yes	yes	no	
D00554	8	Vallières 2017	included	yes	yes	yes	
D02154	112	Zhou 2023	included	yes	yes	no	
D02683	28	Cai 2026	included	yes	yes	no	
D02711	40	Furukawa 2024	included	yes	yes	no	
D02719	78	Peng 2024	included	yes	yes	yes	
D02743	74	Meng 2023	included	yes	yes	no	
D02744	79	Qayyum 2023	included	yes	yes	no	
D02753	65	Ma 2023a	included	yes	yes	no	
D02766	36	Fang 2022	included	yes	yes	no	
D02779	84	Salmanpour 2022a	included	yes	yes	no	
D02802	39	Fontaine 2021	included	yes	yes	no	
D02805	23	Andrearczyk 2021	included	yes	yes	no	
D02806	59	Lv 2021	included	yes	yes	no	
D02847	24	Bizzego 2019	included	yes	yes	no	
D02942	109	Zhang 2026b	included	yes	yes	yes	
D02955	113	Zhou 2026	included	yes	yes	no	
D02964	58	Lin 2026b	included	yes	yes	no	
D02967	33	Cui 2025	included	yes	yes	no	

D02969	48	Hamoush 2025	included	yes	yes	no	
D02973	77	Pan 2023	included	yes	yes	no	
D02975	91	Wang 2022b	included	yes	yes	no	
D02976	81	Saeed 2022	included	yes	yes	no	
D02978	80	Rebaud 2023	included	yes	yes	no	
D02979	72	Meng 2022b	included	yes	yes	no	
D02980	66	Ma 2023b	included	yes	yes	no	
D02981	92	Wang 2022c	included	yes	yes	yes	
D02982	51	Javanmardi 2022	included	yes	yes	no	
D02986	101	Xu 2022	included	yes	yes	no	
D02990	22	Amini 2021	included	yes	yes	no	
D02992	70	Martinez-Larraz 2022	included	yes	yes	no	
D03019	86	Salmanpour 2023c	included	yes	yes	no	
D03028	44	Haddadi Avval 2022	included	yes	yes	no	
D03030	49	Huynh 2022	included	yes	yes	no	
D03032	105	Yousefirizi 2022	included	yes	yes	no	
D04582	87	Starke 2022	included	yes	yes	no	
D04587	85	Salmanpour 2022b	included	yes	yes	no	
D04592	107	Yuan 2022	included	yes	yes	no	
D04603	55	Lang 2022	included	yes	yes	no	
D04606	52	Juanco-Muller 2022	included	yes	yes	no	
D04613	38	Fatan 2022	included	yes	yes	no	
D04616	41	Ghimire 2022	included	yes	yes	no	
D04631	73	Meng 2022c	included	yes	yes	no	
D04632	26	Bourigault 2022	included	yes	yes	no	
D04643	64	Ma 2022	included	yes	yes	no	
D04678	63	Lyu 2023	included	yes	yes	no	
D04709	31	Chen 2023	included	yes	yes	no	
D04749	53	Juanco-Muller 2023	included	yes	yes	no	
D04781	69	Mahdi 2024	included	yes	yes	no	
D04883	37	Farooq 2025	included	yes	yes	no	
D06002	93	Wang 2022d	included	yes	yes	no	
D00098		D00098	excluded (E1)	no	no	no	Only 121 of 391 participants have head and neck malignancy. The protocol note admits a multi-cancer study only if head-and-neck-specific results can be separated; no head-and-neck-only model was fitted, and the separable results do not

							yield the AUC or C-index estimand this review pools.
D00283		D00283	excluded (E9)	no	no	no	Companion conference report of D00156: the same 642 TCIA patients in the same eight centres with the same 507/135 split, and the same FSGCN/FSF analysis. D00156 is the fuller journal report and is retained.
D02778		D02778	excluded (E11)	no	no	no	Substantively eligible on its published abstract, but the SPIE proceedings paper was sought across every generation of this project and never obtained. E11 is a registered full-text exclusion and is applied; an abstract-only record is not admitted.
D04610		D04610	excluded (E3)	no	no	no	Fusion is genuine but upstream, in the segmentation network. The prognostic model itself receives one imaging scalar, tumour volume derived from that segmentation, which is a conventional measure, not a radiomic or learned image feature. The code is the registered E3.
D06001		D06001	excluded (E11)	no	no	no	Substantively eligible on its published abstract, and two reviewers independently included it on that basis, but the article is an Elsevier subscription work with no open version in existence: Unpaywall, OpenAlex, Europe PMC, Semantic Scholar and Crossref all report it closed, with no repository copy and no preprint. E11 applied for consistency with D02778.

Table S3. PROBAST+AI risk of bias, all 100 appraised studies.

D1 participants, D2 predictors, D3 outcome, D4 analysis. Scoring is on positive evidence only: a domain is rated low solely where the report supplies the necessary evidence. Overall risk of bias is high if any domain is high. No overall numerical score is computed, because the instrument does not define one.

**record_id**	**Study**	**1. Participants**	**2. Predictors**	**3. Outcome**	**4. Analysis**	**Overall**
record_id	Study	1. Participants	2. Predictors	3. Outcome	4. Analysis	Overall
D00006	Lin 2026a	High	High	High	High	High
D00010	Zhang 2026a	High	Low	High	High	High
D00057	Ma 2025a	High	Low	Low	High	High
D00058	Ma 2025b	Low	Low	Low	High	High
D00073	Wu 2024a	High	High	Low	High	High
D00084	Oka 2024	High	High	High	High	High
D00102	Wu 2024b	High	High	High	High	High
D00109	Li 2024	High	Low	High	High	High
D00120	De Biase 2024	Low	Low	Unclear	High	High
D00126	Wu 2023	High	High	High	High	High
D00133	Salmanpour 2023a	High	High	High	High	High
D00156	Lv 2023	High	High	High	High	High
D00159	Wang 2023a	Low	High	High	High	High
D00162	Kulanthaivelu 2022	High	High	High	High	High
D00163	Le 2022	High	High	High	High	High
D00165	Zhang 2022	High	High	High	High	High
D00169	Gu 2022	High	High	High	High	High
D00176	Wang 2022a	High	Low	High	High	High
D00178	Meng 2022a	High	Low	High	High	High
D00189	Naser 2022	High	Low	High	High	High
D00227	Chen 2021	High	High	Low	High	High
D00246	Haider 2021	High	High	High	High	High
D00249	Zhong 2021	Low	High	Low	High	High
D00256	Wang 2020	Unclear	High	High	High	High
D00276	Lv 2020	High	Low	High	High	High
D00303	Peng 2019	Low	High	Low	High	High
D00310	Chen 2019	High	High	High	High	High
D00332	Crispin-Ortuzar 2018	High	Low	Low	High	High
D00338	Bogowicz 2017	Low	High	Low	High	High
D00378	Yu 2009	High	High	High	High	High
D00459	Wang 2025	High	High	High	High	High
D00462	Yang 2025	High	High	Low	High	High
D00479	Wang 2024	High	High	High	High	High
D00481	Saikia 2024	High	High	High	High	High
D00485	Wang 2023b	High	High	High	High	High
D00487	Gu 2023	Low	Low	High	High	High
D00488	Xu 2023a	High	Low	High	High	High
D00489	Huynh 2023	Low	Low	Low	High	High
D00494	Xu 2023b	High	Low	High	High	High
D00497	Ding 2023	Low	High	High	High	High
D00498	Starke 2023a	High	Low	High	High	High
D00505	Salmanpour 2023b	High	High	High	High	High
D00507	Salahuddin 2023	High	Low	High	High	High
D00509	Starke 2023b	Low	Low	Low	High	High
D00514	Zhao 2022	Low	High	Low	High	High

D00521	Brancati 2022	High	Low	High	High	High
D00523	Lv 2022	High	Low	Low	High	High
D00534	Haider 2020a	Low	High	High	High	High
D00539	Haider 2020b	High	Low	Unclear	High	High
D00546	Lv Wenbing 2019	High	Low	Low	High	High
D00554	Vallières 2017	High	Low	High	High	High
D02154	Zhou 2023	High	High	Low	High	High
D02683	Cai 2026	High	High	Low	High	High
D02711	Furukawa 2024	High	High	High	High	High
D02719	Peng 2024	High	High	Low	High	High
D02743	Meng 2023	High	Low	Unclear	High	High
D02744	Qayyum 2023	High	High	High	High	High
D02753	Ma 2023a	High	High	Low	High	High
D02766	Fang 2022	High	High	High	High	High
D02802	Fontaine 2021	High	Low	High	High	High
D02805	Andrearczyk 2021	High	Low	Unclear	High	High
D02806	Lv 2021	High	Low	High	High	High
D02847	Bizzego 2019	High	High	High	High	High
D02942	Zhang 2026b	High	Low	High	High	High
D02955	Zhou 2026	High	Low	Low	High	High
D02964	Lin 2026b	High	High	Low	High	High
D02967	Cui 2025	High	High	High	High	High
D02969	Hamoush 2025	High	Low	Low	High	High
D02973	Pan 2023	High	High	Low	High	High
D02975	Wang 2022b	High	High	High	High	High
D02976	Saeed 2022	High	High	Low	High	High
D02978	Rebaud 2023	High	Low	High	High	High
D02979	Meng 2022b	High	Low	High	High	High
D02980	Ma 2023b	High	Low	Low	High	High
D02981	Wang 2022c	High	High	High	High	High
D02982	Javanmardi 2022	High	High	High	High	High
D02986	Xu 2022	High	High	High	High	High
D02990	Amini 2021	High	High	High	High	High
D02992	Martinez-Larraz 2022	High	High	High	High	High
D03019	Salmanpour 2023c	High	High	High	High	High
D03028	Haddadi Avval 2022	High	High	High	High	High
D03030	Huynh 2022	High	High	High	High	High
D03032	Yousefirizi 2022	High	High	High	High	High
D04582	Starke 2022	High	High	High	High	High
D04587	Salmanpour 2022b	High	High	High	High	High
D04592	Yuan 2022	High	High	High	High	High
D04603	Lang 2022	High	High	High	High	High
D04606	Juanco-Muller 2022	High	High	High	High	High
D04613	Fatan 2022	High	High	High	High	High
D04616	Ghimire 2022	High	High	High	High	High
D04631	Meng 2022c	High	High	High	High	High
D04632	Bourigault 2022	High	High	High	High	High
D04643	Ma 2022	High	High	High	High	High
D04678	Lyu 2023	High	High	High	High	High
D04709	Chen 2023	High	Low	High	High	High
D04749	Juanco-Muller 2023	High	High	High	High	High
D04781	Mahdi 2024	High	High	Low	High	High
D04883	Farooq 2025	High	High	High	High	High
D02779	Salmanpour 2022a	High	High	High	High	High

D06002	Wang 2022d	High	High	High	High	High

Table S4. QUADAS-3 assessment, 12 diagnostic estimates.

**record_id**	**Study**	**Task**	**Unit of analysis**	**Participants**	**Index test**	**Target condition**	**Analysis**	**Overall**
record_id	Study	Task	Unit of analysis	Participants	Index test	Target condition	Analysis	Overall
D02766	Fang 2022	hpv16_clade	patients	high	high	low	high	high
D00481	Saikia 2024	hpv_status	patients	high	high	insufficient information	high	high
D00534	Haider 2020a	hpv_status	patients	low	low	high	low	high
D00332	Crispin-Ortuzar 2018	hypoxia	lesions	high	low	high	high	high
D00010	Zhang 2026a	m1_substage	patients	high	high	high	high	high
D00227	Chen 2021	nodal_status_node	lymph_nodes	high	insufficient information	low	high	high
D00310	Chen 2019	nodal_status_node	lymph_nodes	high	high	high	high	high
D02154	Zhou 2023	nodal_status_node	lymph_nodes	high	high	low	high	high
D00521	Brancati 2022	nodal_status_patient	patients	high	high	insufficient information	high	high
D02955	Zhou 2026	nodal_status_patient	patients	high	high	low	high	high
D00462	Yang 2025	pd_l1	patients	high	insufficient information	low	high	high
D00378	Yu 2009	roi_abnormal_vs_normal	regions_of_interest	high	high	high	high	high

Table S5. Sensitivity analyses.

Every estimate is fitted to one analysis per independent cohort lineage. All are random-effects fits with tau-squared by restricted maximum likelihood and a Hartung-Knapp confidence interval. The correlation grid is reported in full: the correlation between a study's two arms is never reported by any study, so the whole grid is shown, and no conclusion rests on a single grid point.

**Analysis**	**Label**	**Sensitivity**	**Independent lineages**	**Contributing analyses**	**Estimate**	**95% CI lower**	**95% CI upper**	**p**	**I-squared %**
Analysis	Label	Sensitivity	Independent lineages	Contributing analyses	Estimate	95% CI lower	95% CI upper	p	I-squared %
A1	Absolute fused prognostic C-index	Primary (one analysis per lineage)	9	16	0.7107	0.6588	0.7626	none	86.1
A1	Absolute fused prognostic C-index	All contributing analyses (dependence NOT modelled)	9	16	0.7162	0.6791	0.7533	7.55e-17	81.1
A1	Absolute fused prognostic C-index	Excluding approximated standard errors	9	9	0.7107	0.6588	0.7626	1.097e-09	86.1
A1	Absolute fused prognostic C-index	External or held-out evaluation only	9	9	0.7107	0.6588	0.7626	1.097e-09	86.1
A2	Absolute fused prognostic AUC	Primary (one analysis per lineage)	7	9	0.8130	0.7325	0.8935	none	56.9
A2	Absolute fused prognostic AUC	All contributing analyses (dependence NOT modelled)	7	9	0.8123	0.7493	0.8752	1.765e-09	51.2
A2	Absolute fused prognostic AUC	Excluding approximated standard errors	4	4	0.8216	0.7085	0.9347	0.0001773	59.8
A2	Absolute fused prognostic AUC	External or held-out evaluation only	5	5	0.8310	0.6974	0.9647	6.616e-05	63.6
D1	Delta C-index, fused vs CT-only	Primary (one analysis per lineage)	6	10	0.0368	0.0017	0.0720	0.0429	0.0
D1	Delta C-index, fused vs CT-only	All contributing analyses (dependence NOT modelled)	6	10	0.0435	0.0209	0.0661	0.001856	0
D1	Delta C-index, fused vs CT-only	Excluding approximated standard errors	6	6	0.0368	0.0017	0.0720	0.0429	0
D1	Delta C-index, fused vs CT-only	External or held-out evaluation only	6	6	0.0368	0.0017	0.0720	0.0429	0
D1	Delta C-index, fused vs CT-only	Assumed correlation 0	6	10	0.0367	0.0009	0.0725	0.04627	0
D1	Delta C-index, fused vs CT-only	Assumed correlation 0.25	6	10	0.0368	0.0012	0.0723	0.04506	0
D1	Delta C-index, fused vs CT-only	Assumed correlation 0.5	6	10	0.0368	0.0017	0.0720	0.0429	0
D1	Delta C-index, fused vs CT-only	Assumed correlation 0.75	6	10	0.0370	0.0030	0.0710	0.03802	0
D1	Delta C-index, fused vs CT-only	Assumed correlation 0.9	6	10	0.0331	-0.0052	0.0714	0.07717	51.4
D2	Delta C-index, fused vs PET-only	Primary (one analysis per lineage)	6	10	0.0422	0.0143	0.0701	0.0115	0.0
D2	Delta C-index, fused vs PET-only	All contributing analyses (dependence NOT modelled)	6	10	0.0397	0.0129	0.0664	0.00853	0

D2	Delta C-index, fused vs PET-only	Excluding approximated standard errors	6	6	0.0422	0.0143	0.0701	0.0115	0
D2	Delta C-index, fused vs PET-only	External or held-out evaluation only	6	6	0.0422	0.0143	0.0701	0.0115	0
D2	Delta C-index, fused vs PET-only	Assumed correlation 0	6	10	0.0424	0.0144	0.0703	0.0114	0
D2	Delta C-index, fused vs PET-only	Assumed correlation 0.25	6	10	0.0423	0.0144	0.0702	0.01143	0
D2	Delta C-index, fused vs PET-only	Assumed correlation 0.5	6	10	0.0422	0.0143	0.0701	0.0115	0
D2	Delta C-index, fused vs PET-only	Assumed correlation 0.75	6	10	0.0419	0.0136	0.0702	0.01256	3.5
D2	Delta C-index, fused vs PET-only	Assumed correlation 0.9	6	10	0.0420	0.0118	0.0722	0.01596	59.4
D3	Delta C-index, fused vs clinical-only	Primary (one analysis per lineage)	7	8	0.0914	0.0222	0.1606	0.01787	79.0
D3	Delta C-index, fused vs clinical-only	All contributing analyses (dependence NOT modelled)	7	8	0.0708	-0.0017	0.1433	0.05436	83
D3	Delta C-index, fused vs clinical-only	Excluding approximated standard errors	7	7	0.0914	0.0222	0.1606	0.01787	79
D3	Delta C-index, fused vs clinical-only	External or held-out evaluation only	7	7	0.0914	0.0222	0.1606	0.01787	79
D3	Delta C-index, fused vs clinical-only	Assumed correlation 0	7	8	0.1000	0.0322	0.1678	0.01122	59.2
D3	Delta C-index, fused vs clinical-only	Assumed correlation 0.25	7	8	0.0962	0.0279	0.1646	0.01371	69.1
D3	Delta C-index, fused vs clinical-only	Assumed correlation 0.5	7	8	0.0914	0.0222	0.1606	0.01787	79
D3	Delta C-index, fused vs clinical-only	Assumed correlation 0.75	7	8	0.0849	0.0146	0.1552	0.02543	88.9
D3	Delta C-index, fused vs clinical-only	Assumed correlation 0.9	7	8	0.0799	0.0091	0.1508	0.03288	94.9

Table S6. METRICS v1.0 reporting completeness.

Response counts for the thirty items. No total score is computed, because the instrument does not define one, and conditionality is enforced in both directions, so an item which cannot apply to a design is recorded as not applicable.

record_id	Study	Yes	No	Not applicable	Unscoreable	Applicability
record_id	Study	Yes	No	Not applicable	Unscoreable	Applicability
D00006	Lin 2026a	19	9	2	0	handcrafted_features;tabular_data
D00010	Zhang 2026a	15	13	2	0	handcrafted_features;tabular_data
D00057	Ma 2025a	19	6	5	0	end_to_end_deep_learning
D00058	Ma 2025b	18	8	4	0	end_to_end_deep_learning;automated_segmentation
D00073	Wu 2024a	11	14	5	0	end_to_end_deep_learning
D00084	Oka 2024	11	17	2	0	handcrafted_features;tabular_data
D00102	Wu 2024b	15	10	5	0	end_to_end_deep_learning
D00109	Li 2024	15	13	2	0	handcrafted_features;tabular_data
D00120	De Biase 2024	18	8	4	0	end_to_end_deep_learning;automated_segmentation
D00126	Wu 2023	16	12	2	0	handcrafted_features;tabular_data
D00133	Salmanpour 2023a	10	18	2	0	handcrafted_features;tabular_data
D00156	Lv 2023	21	7	2	0	handcrafted_features;tabular_data
D00159	Wang 2023a	18	10	2	0	handcrafted_features;tabular_data
D00162	Kulanthaivelu 2022	14	14	2	0	handcrafted_features;tabular_data
D00163	Le 2022	18	10	2	0	handcrafted_features;tabular_data
D00165	Zhang 2022	16	12	2	0	handcrafted_features;tabular_data
D00169	Gu 2022	19	10	1	0	handcrafted_features;tabular_data;end_to_end_deep_learning
D00176	Wang 2022a	13	12	5	0	end_to_end_deep_learning
D00178	Meng 2022a	17	13	0	0	handcrafted_features;tabular_data;end_to_end_deep_learning;automated_segmentation
D00189	Naser 2022	14	14	2	0	tabular_data;end_to_end_deep_learning
D00227	Chen 2021	8	21	1	0	handcrafted_features;tabular_data;end_to_end_deep_learning
D00246	Haider 2021	19	9	2	0	handcrafted_features;tabular_data
D00249	Zhong 2021	18	10	2	0	handcrafted_features;tabular_data
D00256	Wang 2020	16	12	2	0	handcrafted_features;tabular_data
D00276	Lv 2020	20	8	2	0	handcrafted_features;tabular_data
D00303	Peng 2019	21	6	2	1	handcrafted_features;tabular_data
D00310	Chen 2019	7	22	1	0	handcrafted_features;tabular_data;end_to_end_deep_learning
D00332	Crispin-Ortuzar 2018	16	12	2	0	handcrafted_features;tabular_data
D00338	Bogowicz 2017	16	13	1	0	handcrafted_features;tabular_data;automated_segmentation
D00378	Yu 2009	14	14	2	0	handcrafted_features;tabular_data
D00459	Wang 2025	14	15	1	0	handcrafted_features;tabular_data;end_to_end_deep_learning
D00462	Yang 2025	17	11	2	0	handcrafted_features;tabular_data
D00479	Wang 2024	11	14	5	0	end_to_end_deep_learning
D00481	Saikia 2024	13	12	5	0	end_to_end_deep_learning
D00485	Wang 2023b	16	12	2	0	handcrafted_features;tabular_data
D00487	Gu 2023	24	6	0	0	handcrafted_features;tabular_data;end_to_end_deep_learning;automated_segmentation
D00488	Xu 2023a	21	7	2	0	handcrafted_features;tabular_data
D00489	Huynh 2023	19	10	1	0	handcrafted_features;tabular_data;end_to_end_deep_learning
D00494	Xu 2023b	25	4	1	0	handcrafted_features;tabular_data;automated_segmentation
D00497	Ding 2023	17	11	2	0	handcrafted_features;tabular_data
D00498	Starke 2023a	15	10	5	0	end_to_end_deep_learning
D00505	Salmanpour 2023b	8	21	1	0	handcrafted_features;tabular_data;end_to_end_deep_learning
D00507	Salahuddin 2023	22	7	1	0	handcrafted_features;tabular_data;automated_segmentation
D00509	Starke 2023b	19	9	2	0	handcrafted_features;tabular_data
D00514	Zhao 2022	14	11	5	0	end_to_end_deep_learning

D00521	Brancati 2022	16	12	2	0	handcrafted_features;tabular_data
D00523	Lv 2022	20	8	2	0	handcrafted_features;tabular_data
D00534	Haider 2020a	20	8	2	0	handcrafted_features;tabular_data
D00539	Haider 2020b	16	12	2	0	handcrafted_features;tabular_data
D00546	Lv Wenbing 2019	17	11	2	0	handcrafted_features;tabular_data
D00554	Vallières 2017	20	8	2	0	handcrafted_features;tabular_data
D02154	Zhou 2023	8	20	2	0	handcrafted_features;tabular_data
D02683	Cai 2026	17	11	2	0	handcrafted_features;tabular_data
D02711	Furukawa 2024	15	13	2	0	handcrafted_features;tabular_data
D02719	Peng 2024	21	7	2	0	handcrafted_features;tabular_data
D02743	Meng 2023	17	13	0	0	handcrafted_features;tabular_data;end_to_end_deep_learning;automated_segmentation
D02744	Qayyum 2023	14	15	1	0	handcrafted_features;tabular_data;automated_segmentation
D02753	Ma 2023a	15	10	5	0	end_to_end_deep_learning
D02766	Fang 2022	10	19	1	0	handcrafted_features;tabular_data;end_to_end_deep_learning
D02802	Fontaine 2021	17	12	1	0	handcrafted_features;tabular_data;automated_segmentation
D02805	Andrearczyk 2021	15	15	0	0	handcrafted_features;tabular_data;end_to_end_deep_learning;automated_segmentation
D02806	Lv 2021	21	7	2	0	handcrafted_features;tabular_data
D02847	Bizzego 2019	12	17	1	0	handcrafted_features;tabular_data;end_to_end_deep_learning
D02942	Zhang 2026b	16	12	2	0	tabular_data;end_to_end_deep_learning
D02955	Zhou 2026	11	19	0	0	handcrafted_features;tabular_data;end_to_end_deep_learning;automated_segmentation
D02964	Lin 2026b	18	10	2	0	handcrafted_features;tabular_data
D02967	Cui 2025	9	17	4	0	end_to_end_deep_learning;automated_segmentation
D02969	Hamoush 2025	14	14	2	0	handcrafted_features;tabular_data
D02973	Pan 2023	17	11	2	0	handcrafted_features;tabular_data
D02975	Wang 2022b	20	9	1	0	handcrafted_features;tabular_data;automated_segmentation
D02976	Saeed 2022	14	14	2	0	tabular_data;end_to_end_deep_learning
D02978	Rebaud 2023	20	9	1	0	handcrafted_features;tabular_data;automated_segmentation
D02979	Meng 2022b	19	11	0	0	handcrafted_features;tabular_data;end_to_end_deep_learning;automated_segmentation
D02980	Ma 2023b	22	8	0	0	handcrafted_features;tabular_data;end_to_end_deep_learning;automated_segmentation
D02981	Wang 2022c	11	17	2	0	tabular_data;end_to_end_deep_learning
D02982	Javanmardi 2022	4	21	5	0	end_to_end_deep_learning
D02986	Xu 2022	20	9	1	0	handcrafted_features;tabular_data;automated_segmentation
D02990	Amini 2021	13	15	2	0	handcrafted_features;tabular_data
D02992	Martinez-Larraz 2022	10	19	1	0	handcrafted_features;tabular_data;automated_segmentation
D03019	Salmanpour 2023c	10	18	2	0	tabular_data;automated_segmentation
D03028	Haddadi Avval 2022	16	12	2	0	handcrafted_features;tabular_data
D03030	Huynh 2022	19	10	1	0	handcrafted_features;tabular_data;end_to_end_deep_learning
D03032	Yousefirizi 2022	14	16	0	0	handcrafted_features;tabular_data;end_to_end_deep_learning;automated_segmentation
D04582	Starke 2022	19	10	1	0	handcrafted_features;tabular_data;automated_segmentation
D04587	Salmanpour 2022b	14	15	1	0	handcrafted_features;tabular_data;automated_segmentation
D04592	Yuan 2022	18	11	1	0	handcrafted_features;tabular_data;automated_segmentation
D04603	Lang 2022	15	11	4	0	end_to_end_deep_learning;automated_segmentation
D04606	Juanco-Muller 2022	15	14	1	0	handcrafted_features;tabular_data;automated_segmentation
D04613	Fatan 2022	12	17	1	0	handcrafted_features;tabular_data;automated_segmentation
D04616	Ghimire 2022	12	18	0	0	handcrafted_features;tabular_data;end_to_end_deep_learning;automated_segmentation
D04631	Meng 2022c	14	12	4	0	end_to_end_deep_learning;automated_segmentation
D04632	Bourigault 2022	16	13	1	0	handcrafted_features;tabular_data;automated_segmentation
D04643	Ma 2022	15	12	3	0	tabular_data
D04678	Lyu 2023	13	16	1	0	handcrafted_features;tabular_data;automated_segmentation
D04709	Chen 2023	13	16	1	0	handcrafted_features;tabular_data;automated_segmentation
D04749	Juanco-Muller 2023	17	13	0	0	handcrafted_features;tabular_data;end_to_end_deep_learning;automated_segmentation
D04781	Mahdi 2024	10	18	2	0	handcrafted_features;tabular_data
D04883	Farooq 2025	6	19	5	0	end_to_end_deep_learning
D02779	Salmanpour 2022a	9	19	2	0	

D06002	Wang 2022d	12	14	4	0	

Supplementary Figures

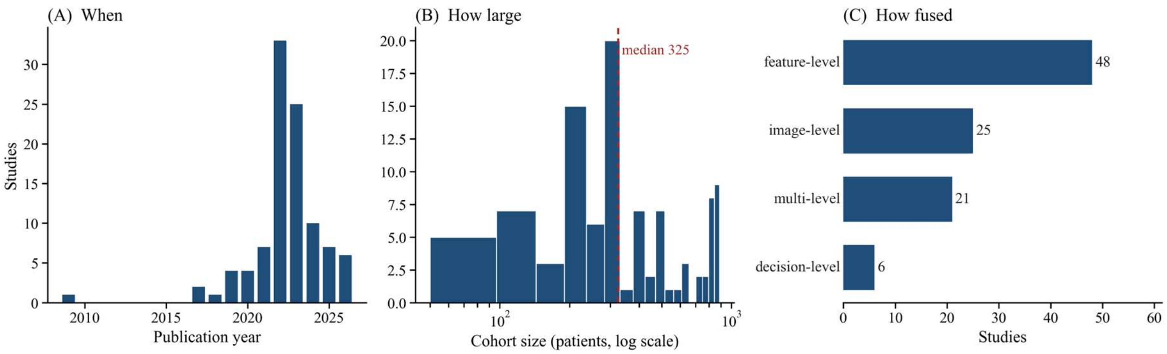

Figure S1. The included studies described: (A) publication year, (B) cohort size on a logarithmic scale with the median marked, (C) how the two modalities were combined.

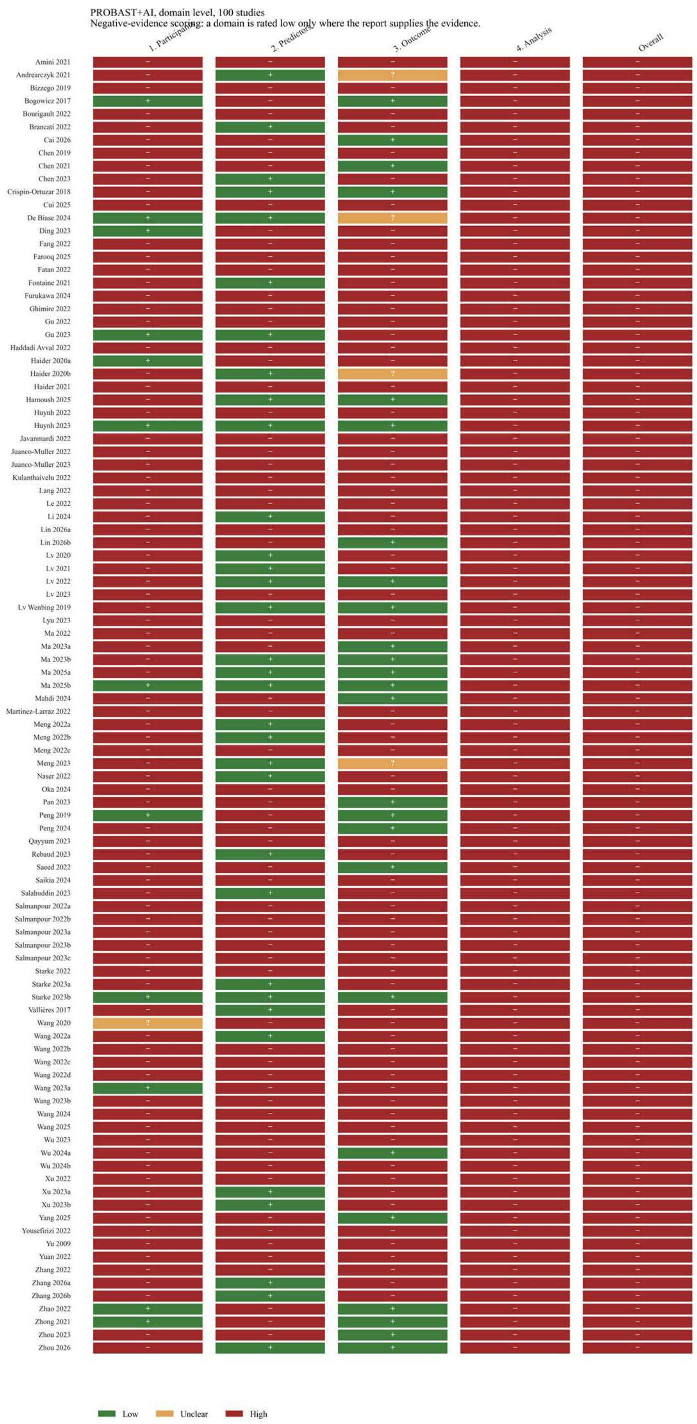

PROBAST+AI, domain level, 100 studies
Negative-evidence scoring: a domain is rated low only where the report supplies the evidence.
1. Participants
2. Predictors
3. Outcome
4. Analysis
Overall
Amini 2021
Andrearczyk 2021
Bizzego 2019
Bogowicz 2017
Bourigault 2022
Brancati 2022
Cai 2026
Chen 2019
Chen 2021
Chen 2023
Crispin-Ortuzar 2018
Cui 2025
De Biase 2024
Ding 2023
Fang 2022
Farooq 2025
Fatan 2022
Fontaine 2021
Furukawa 2024
Ghimire 2022
Gu 2022
Gu 2023
Haddadi Avval 2022
Haider 2020a
Haider 2020b
Haider 2021
Hamoush 2025
Huynh 2022
Huynh 2023
Javanmardi 2022
Juanco-Muller 2022
Juanco-Muller 2023
Kulanthaivelu 2022
Lang 2022
Le 2022
Li 2024
Lin 2026a
Lin 2026b
Lv 2020
Lv 2021
Lv 2022
Lv 2023
Lv Wenbing 2019
Lyu 2023
Ma 2022
Ma 2023a
Ma 2023b
Ma 2025a
Ma 2025b
Mahdi 2024
Martinez-Larraz 2022
Meng 2022a
Meng 2022b
Meng 2022c
Meng 2023
Naser 2022
Oka 2024
Pan 2023
Peng 2019
Peng 2024
Qayyum 2023
Rebaud 2023
Saeed 2022
Saikia 2024
Salahuddin 2023
Salmanpour 2022a
Salmanpour 2022b
Salmanpour 2023a
Salmanpour 2023b
Salmanpour 2023c
Starke 2022
Starke 2023a
Starke 2023b
Vallières 2017
Wang 2020
Wang 2022a
Wang 2022b
Wang 2022c
Wang 2022d
Wang 2023a
Wang 2023b
Wang 2024
Wang 2025
Wu 2023
Wu 2024a
Wu 2024b
Xu 2022
Xu 2023a
Xu 2023b
Yang 2025
Yousefirizi 2022
Yu 2009
Yuan 2022
Zhang 2022
Zhang 2026a
Zhang 2026b
Zhao 2022
Zhong 2021
Zhou 2023
Zhou 2026
Low
Unclear
High

Figure S2. PROBAST+AI risk of bias, domain by domain, for every appraised study. Negative-evidence scoring: a domain is rated low only where the report supplies the evidence.

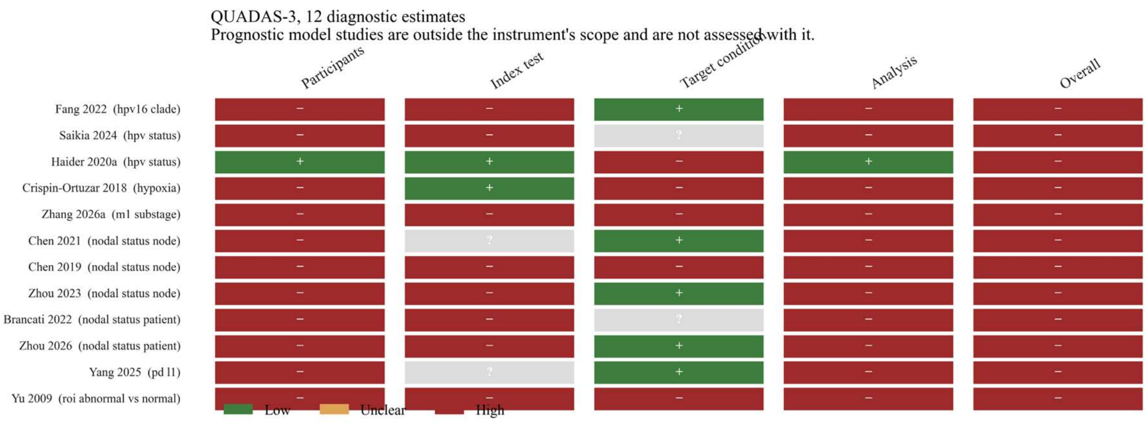

Figure S3. QUADAS-3 for the diagnostic estimates. Prognostic model studies are outside the instrument's scope and are not assessed with it.

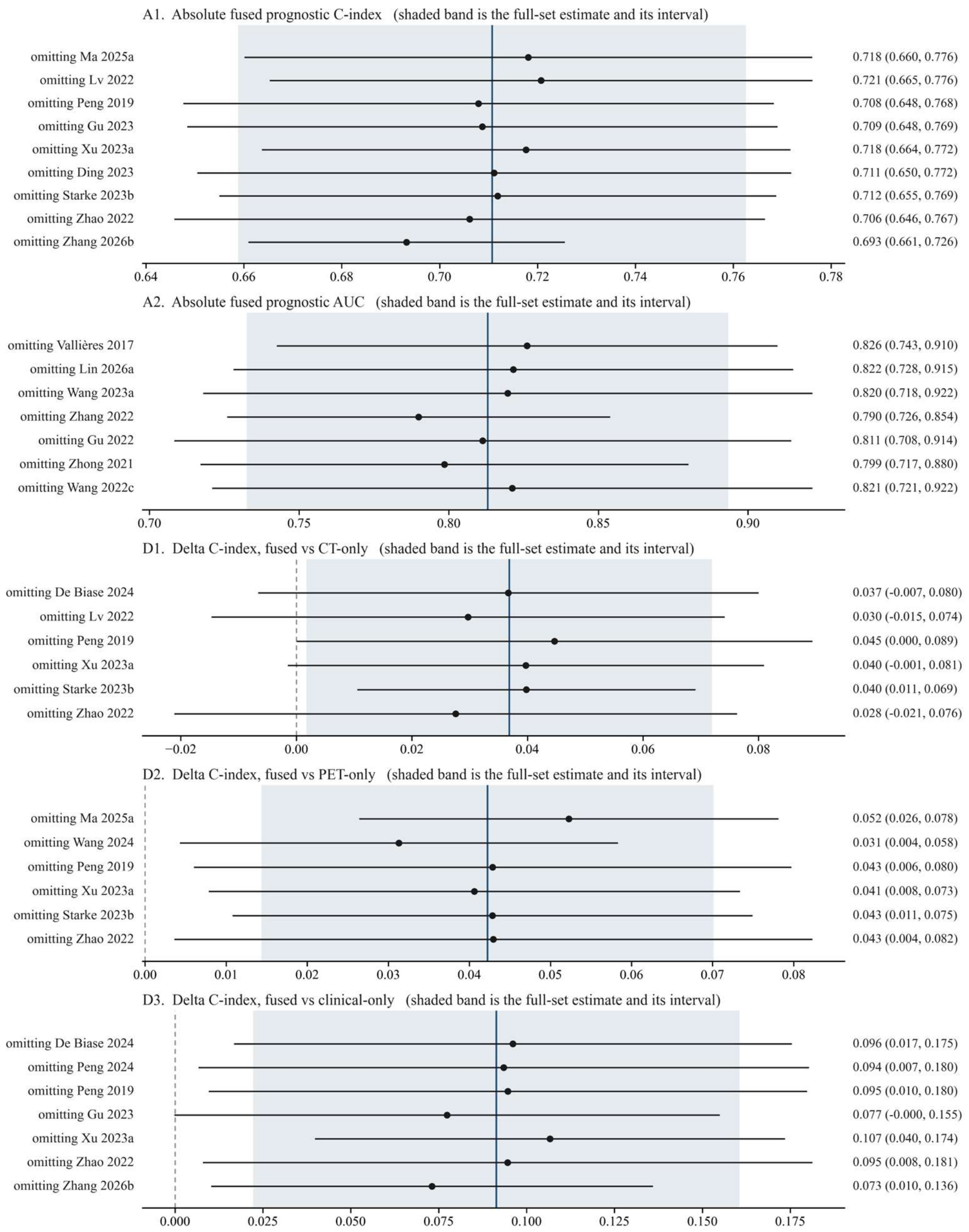

A1. Absolute fused prognostic C-index (shaded band is the full-set estimate and its interval)
omitting Ma 2025a
omitting Lv 2022
omitting Peng 2019
omitting Gu 2023
omitting Xu 2023a
omitting Ding 2023
omitting Starke 2023b
omitting Zhao 2022
omitting Zhang 2026b
0.718 (0.660, 0.776)
0.721 (0.665, 0.776)
0.708 (0.648, 0.768)
0.709 (0.648, 0.769)
0.718 (0.664, 0.772)
0.711 (0.650, 0.772)
0.712 (0.655, 0.769)
0.706 (0.646, 0.767)
0.693 (0.661, 0.726)
0.64 0.66 0.68 0.70 0.72 0.74 0.76 0.78
A2. Absolute fused prognostic AUC (shaded band is the full-set estimate and its interval)
omitting Vallières 2017
omitting Lin 2026a
omitting Wang 2023a
omitting Zhang 2022
omitting Gu 2022
omitting Zhong 2021
omitting Wang 2022c
0.826 (0.743, 0.910)
0.822 (0.728, 0.915)
0.820 (0.718, 0.922)
0.790 (0.726, 0.854)
0.811 (0.708, 0.914)
0.799 (0.717, 0.880)
0.821 (0.721, 0.922)
0.70 0.75 0.80 0.85 0.90
D1. Delta C-index, fused vs CT-only (shaded band is the full-set estimate and its interval)
omitting De Biase 2024
omitting Lv 2022
omitting Peng 2019
omitting Xu 2023a
omitting Starke 2023b
omitting Zhao 2022
0.037 (-0.007, 0.080)
0.030 (-0.015, 0.074)
0.045 (0.000, 0.089)
0.040 (-0.001, 0.081)
0.040 (0.011, 0.069)
0.028 (-0.021, 0.076)
−0.02 0.00 0.02 0.04 0.06 0.08
D2. Delta C-index, fused vs PET-only (shaded band is the full-set estimate and its interval)
omitting Ma 2025a
omitting Wang 2024
omitting Peng 2019
omitting Xu 2023a
omitting Starke 2023b
omitting Zhao 2022
0.052 (0.026, 0.078)
0.031 (0.004, 0.058)
0.043 (0.006, 0.080)
0.041 (0.008, 0.073)
0.043 (0.011, 0.075)
0.043 (0.004, 0.082)
0.00 0.01 0.02 0.03 0.04 0.05 0.06 0.07 0.08
D3. Delta C-index, fused vs clinical-only (shaded band is the full-set estimate and its interval)
omitting De Biase 2024
omitting Peng 2024
omitting Peng 2019
omitting Gu 2023
omitting Xu 2023a
omitting Zhao 2022
omitting Zhang 2026b
0.096 (0.017, 0.175)
0.094 (0.007, 0.180)
0.095 (0.010, 0.180)
0.077 (-0.000, 0.155)
0.107 (0.040, 0.174)
0.095 (0.008, 0.181)
0.073 (0.010, 0.136)
0.000 0.025 0.050 0.075 0.100 0.125 0.150 0.175

Figure S4. Leave-one-out for every pooled analysis, one row per omitted cohort lineage. The shaded band is the full-set estimate with its confidence interval.

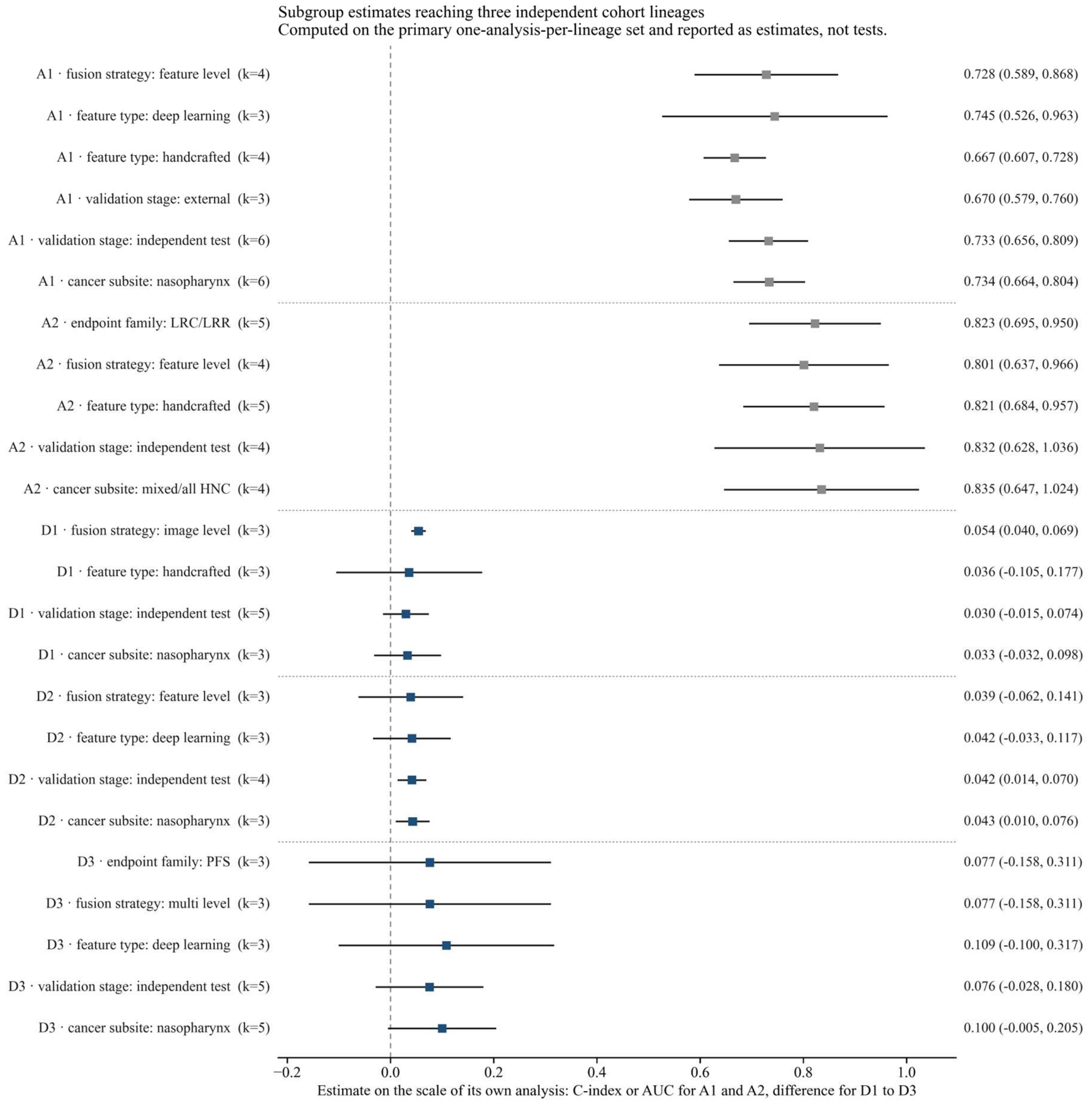

Subgroup estimates reaching three independent cohort lineages
Computed on the primary one-analysis-per-lineage set and reported as estimates, not tests.
A1 · fusion strategy: feature level (k=4)
A1 · feature type: deep learning (k=3)
A1 · feature type: handcrafted (k=4)
A1 · validation stage: external (k=3)
A1 · validation stage: independent test (k=6)
A1 · cancer subsite: nasopharynx (k=6)
A2 · endpoint family: LRC/LRR (k=5)
A2 · fusion strategy: feature level (k=4)
A2 · feature type: handcrafted (k=5)
A2 · validation stage: independent test (k=4)
A2 · cancer subsite: mixed/all HNC (k=4)
D1 · fusion strategy: image level (k=3)
D1 · feature type: handcrafted (k=3)
D1 · validation stage: independent test (k=5)
D1 · cancer subsite: nasopharynx (k=3)
D2 · fusion strategy: feature level (k=3)
D2 · feature type: deep learning (k=3)
D2 · validation stage: independent test (k=4)
D2 · cancer subsite: nasopharynx (k=3)
D3 · endpoint family: PFS (k=3)
D3 · fusion strategy: multi level (k=3)
D3 · feature type: deep learning (k=3)
D3 · validation stage: independent test (k=5)
D3 · cancer subsite: nasopharynx (k=5)
0.728 (0.589, 0.868)
0.745 (0.526, 0.963)
0.667 (0.607, 0.728)
0.670 (0.579, 0.760)
0.733 (0.656, 0.809)
0.734 (0.664, 0.804)
0.823 (0.695, 0.950)
0.801 (0.637, 0.966)
0.821 (0.684, 0.957)
0.832 (0.628, 1.036)
0.835 (0.647, 1.024)
0.054 (0.040, 0.069)
0.036 (-0.105, 0.177)
0.030 (-0.015, 0.074)
0.033 (-0.032, 0.098)
0.039 (-0.062, 0.141)
0.042 (-0.033, 0.117)
0.042 (0.014, 0.070)
0.043 (0.010, 0.076)
0.077 (-0.158, 0.311)
0.077 (-0.158, 0.311)
0.109 (-0.100, 0.317)
0.076 (-0.028, 0.180)
0.100 (-0.005, 0.205)
−0.2
0.0
0.2
0.4
0.6
0.8
1.0
Estimate on the scale of its own analysis: C-index or AUC for A1 and A2, difference for D1 to D3

Figure S5. Every subgroup estimate reaching three independent cohort lineages, computed on the primary one-analysis-per-lineage set and reported as an estimate; no between-level test is performed.

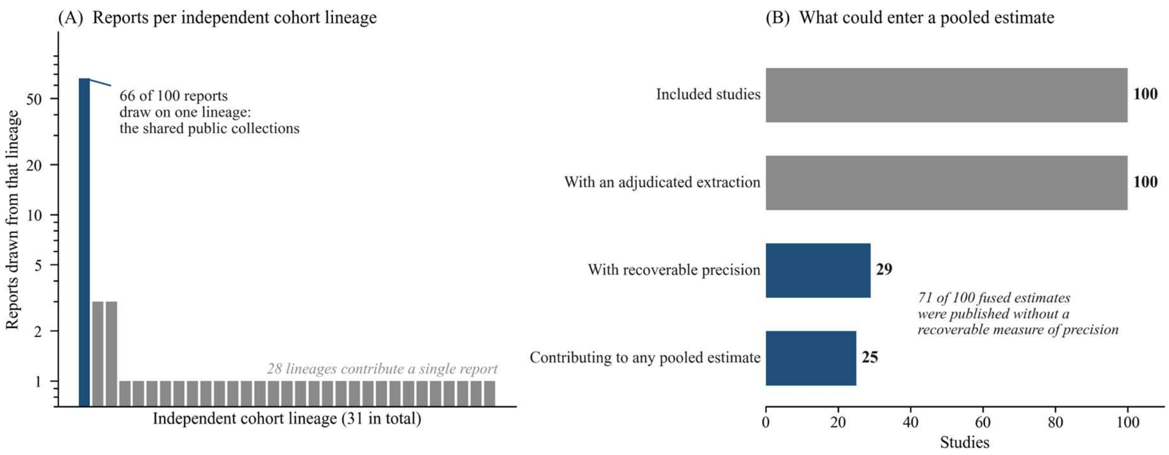

Figure S6. Why the included reports are not that many independent pieces of evidence. (A) Reports drawn from each independent cohort lineage, on a logarithmic scale. (B) How many studies could enter a pooled estimate, and where the rest were lost.

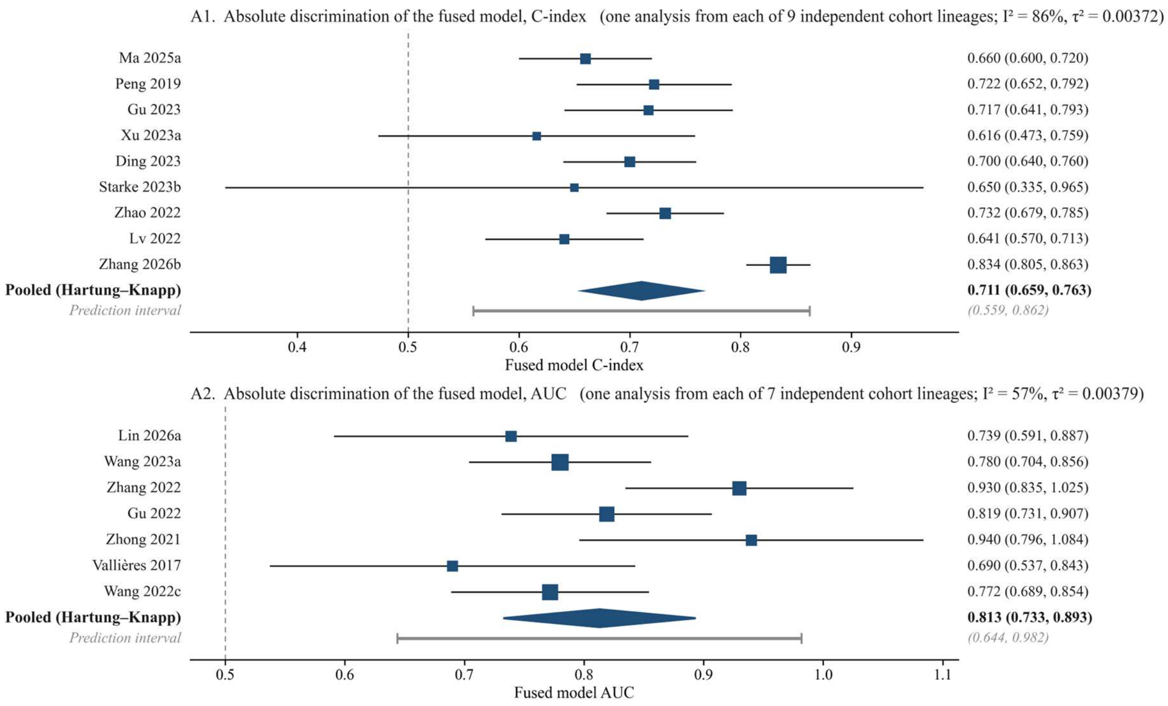

Figure S7. Absolute discrimination of the fused model, concordance index (A1) and area under the curve (A2), one analysis from each independent cohort lineage.

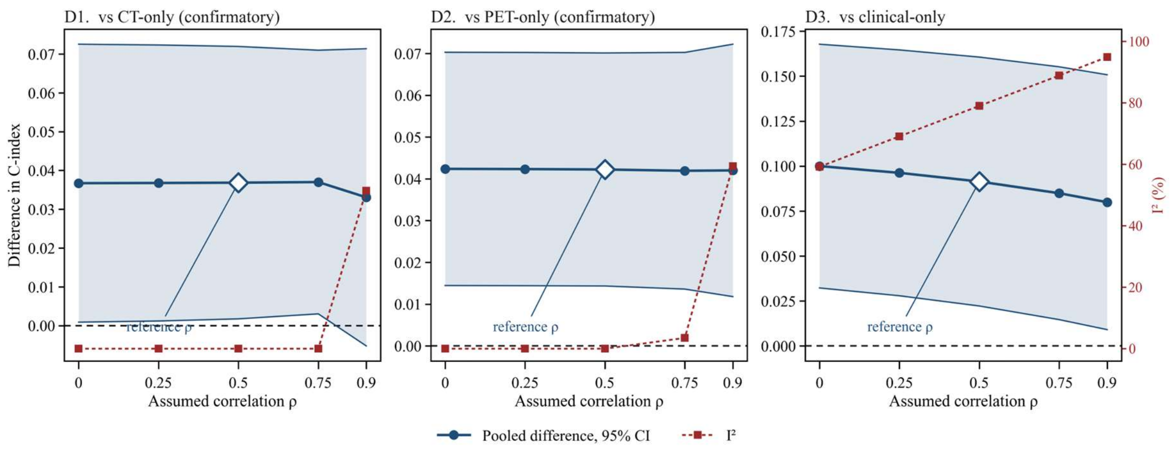

Figure S8. Each difference across the grid of assumed correlations between the paired arms, with the heterogeneity that accompanies each assumption.

Table S7. The completed PRISMA 2020 checklist.

Each item points at the section of the manuscript that reports it. Where an item does not apply, the entry says so and gives the reason.

Item	Checklist item	Reported in
1	Title	Title page: identified as a systematic review and meta-analysis.
2	Abstract	Abstract: structured, reporting objective, design, sources, eligibility, synthesis, results with intervals, and conclusions.
3	Rationale	Introduction, paragraphs 1 to 5.
4	Objectives	Introduction, final paragraph.
5	Eligibility criteria	Methods 2.1.
6	Information sources	Methods 2.2; Table S1, with interface and yield per source.
7	Search strategy	The full strategies, reproduced after Table S9.
8	Selection process	Methods 2.3: two independent reviewers at every stage, disagreements resolved against the source document.
9	Data collection process	Methods 2.4: independent duplicate extraction, adjudicated field by field.
10a	Data items: outcomes	Methods 2.4 and 2.6.
10b	Data items: other variables	Methods 2.4; workbook sheet S1_characteristics.
11	Study risk of bias assessment	Methods 2.5; Results 3.8; Tables S3, S4 and S6; Figures S2 and S3.
12	Effect measures	Methods 2.6: the within-study difference in discrimination, with Somers' D alongside.
13a	Synthesis: eligibility for synthesis	Methods 2.6; workbook sheet S4_pool_membership.
13b	Synthesis: data preparation	Methods 2.6; workbook sheet S10_derived_standard_errors.
13c	Synthesis: tabulation and visual display	Table 1; Figure 2.
13d	Synthesis: methods	Methods 2.6: random effects, restricted maximum likelihood, Hartung-Knapp intervals, one analysis per lineage.
13e	Synthesis: heterogeneity investigation	Methods 2.6; Results 3.7 and 3.9; Table S5.
13f	Synthesis: sensitivity analyses	Results 3.9; Table S5; Figures S4 and S8.
14	Reporting bias assessment	Methods 2.6 and Results 3.10: no analysis reaches the ten independent contributions the protocol requires before a funnel plot or a test of asymmetry is interpretable, so none is performed, and this is stated.
15	Certainty assessment	Results 3.8, closing paragraph; Discussion 4.4.
16a	Results: study selection	Results 3.1; Figure 1; workbook sheet S11_PRISMA_by_wave.
16b	Results: studies excluded with reasons	Figure 1 and workbook sheet S11_PRISMA_by_wave: full-text exclusions by wave and registered code; Table S2: the corpus, with each report removed at final revalidation under its code and reason.
17	Study characteristics	Results 3.2 to 3.4; workbook sheet S1_characteristics.
18	Risk of bias in studies	Results 3.8; Figure 3; Tables S3, S4 and S6.
19	Results of individual studies	Workbook sheets S2_performance_and_comparators and S3_direction_of_effect.
20a	Results of syntheses: characteristics and risk of bias	Results 3.2 and 3.8.
20b	Results of syntheses: statistical results	Results 3.5 and 3.6; Table 1; Figure 2.
20c	Results of syntheses: heterogeneity	Results 3.5 to 3.7; Table 1, with I-squared, its interval, tau-squared and a prediction interval.
20d	Results of syntheses: sensitivity	Results 3.9; Table S5; Figure S4.
21	Reporting biases	Results 3.10; Discussion 4.5.
22	Certainty of evidence	Results 3.8, closing paragraph; Discussion 4.4.
23a	Discussion: interpretation	Discussion 4.1 and 4.2.
23b	Discussion: limitations of the evidence	Discussion 4.4.
23c	Discussion: limitations of the review	Discussion 4.5.
23d	Discussion: implications	Discussion 4.6.
24a	Registration and protocol	Methods, opening paragraph: PROSPERO CRD420261319855.

24b	Registration: where the protocol can be accessed	PROSPERO record CRD420261319855.
24c	Amendments to the protocol	Table S8, with every deviation and its reason, and the PROSPERO amendment.
25	Support	Funding statement.
26	Competing interests	Conflicts of Interest statement.
27	Availability of data, code and other materials	Data Availability Statement; the supplementary data workbook.

Table S8. Deviations from the registered protocol.

Every departure from the registered plan, with the reason for it. The amendment text is filed with the review data.

Item	As registered	As conducted	Reason
Sources searched	PubMed/MEDLINE, CENTRAL through the Cochrane Library, ASSIA through ProQuest, Embase, Scopus	Web of Science Core Collection, IEEE Xplore, OpenAlex and Crossref added. Embase and Scopus not searched: authenticated access attempts returned a negative entitlement on both platforms, and no unofficial route was used.	Broadens coverage. The two unsearched platforms are a standing limitation, reported as one.
Unit of analysis	the study	the independent patient lineage, keyed to the cohort on which the estimate is evaluated	Reports sharing patients are not independent replications; counting them as such would produce intervals that are too narrow.
Result selection within a lineage	not specified beyond one result per study	one analysis per lineage, by largest independently held-out evaluation set, then earliest publication year, then record identifier	Fixed in the statistical analysis plan before any result was seen; the rule never consults the effect, verified mechanically by re-running selection with every difference negated.
Risk-of-bias instrument	PROBAST	PROBAST+AI	The models under review are predominantly machine-learning models, which the AI extension was written for.
Reporting-quality instrument	a study-specific scoring scheme	METRICS v1.0, applied item by item with no total score	A validated instrument replaces an unvalidated one. METRICS defines no total, so none is computed.
Diagnostic accuracy instrument	QUADAS-2	QUADAS-3	QUADAS-3 was released in February 2026 and supersedes QUADAS-2 for this application.
Multiplicity	not specified	a confirmatory family of exactly two hypotheses (fused versus CT-only and fused versus PET-only on the concordance index), Bonferroni two-sided alpha 0.025, with 97.5% intervals reported alongside 95% intervals	The family was specified in the statistical analysis plan, which was finalised and dated before data extraction for the enlarged evidence base began. Every other estimate is reported for estimation and no p value from it is compared to any threshold.
Minimum for synthesis	three studies	three independent lineages generally; five commensurate paired comparisons for contrasts on the area under the curve	The stricter metric-specific minimum was fixed in the analysis plan before extraction opened. It is why the three area-under-the-curve difference analyses are reported individually and not pooled.
Paired variance	not specified	Var(delta) = SE_fused^2 + SE_comparator^2 - 2 rho SE_fused SE_comparator, across a grid of rho in {0, 0.25, 0.50, 0.75, 0.90} with 0.50 as the reference	The two arms are measured on the same patients. The correlation is never reported, so the whole grid is examined and the sensitivity of every conclusion to it is stated.
Standardized effect	not specified	Somers' D, D = 2C - 1, reported alongside every difference in the concordance index	An exact linear re-expression that assumes nothing. A standardized mean difference is not computed, because no between-subject standard deviation exists to divide by.

Table S9. Guide to the supplementary data workbook.

The workbook accompanying this article carries every per-study row behind the review. A reader who wants to check any figure in the paper against the studies that produced it will find them here.

Sheet	Rows	What it contains
S1_characteristics	100	one row per included study: population, task, pipeline, lineage
S0_corpus	105	the review corpus: each included study, its denominators, and every report removed at final revalidation
S2_performance_and_comparators	400	every extracted value with its locator in the source report, and the comparator audit class for each pair

S3_direction_of_effect	6	the direction of every valid paired comparison, counted per lineage and per contributing analysis
S4_pool_membership	65	which studies entered which analysis, and the representative chosen
S5_PROBAST_AI	100	risk of bias by domain, negative-evidence scoring
S6_METRICS_v1	100	reporting completeness, response counts, no total score
S7_QUADAS_3	12	diagnostic accuracy appraisal for the diagnostic estimates
S8_sensitivity	35	every sensitivity analysis of the analysis plan, including the correlation grid
S9_leave_one_out	35	leave-one-out results, one row per omitted lineage
S10_derived_standard_errors	22	every standard error derived under tier 3, with the class frequencies used, and every derivation refused with its reason
S11_PRISMA_by_wave	63	the flow decomposed by search wave; the merged totals are their sums

Full search strategies, verbatim

Each string below is the one pasted into the platform, reproduced without alteration, for every source whose executed string was retained. Sources searched programmatically, and the one platform search whose exact string was not retained, are listed after them and are not reconstructed.

PubMed/MEDLINE - 30 July 2026; updated 17 August 2026; retrieved 1,232 (delta 6). Interface: NCBI E-utilities and the web interface; no filters, no date limit.

```
# PubMed Search Strategy

# Systematic Review: Radiomics Using Fused PET and CT Data in Head and Neck Cancer

# PROSPERO: CRD420261319855

# Date: 2026-03-27

# Version: 3.3 (refined from v3.2 — tightened Block 3, added fusion block)



("Head and Neck Neoplasms"[MeSH Terms] OR

"head and neck cancer"[tiab] OR "head and neck cancers"[tiab] OR

"HNSCC"[tiab] OR "HNC"[tiab] OR

"oropharyngeal cancer"[tiab] OR "oropharyngeal carcinoma"[tiab] OR

"laryngeal cancer"[tiab] OR "laryngeal carcinoma"[tiab] OR

"hypopharyngeal cancer"[tiab] OR "hypopharyngeal carcinoma"[tiab] OR

"nasopharyngeal cancer"[tiab] OR "nasopharyngeal carcinoma"[tiab] OR "NPC"[tiab] OR

"oral cavity cancer"[tiab] OR "oral cancer"[tiab] OR

"pharyngeal cancer"[tiab] OR "pharyngeal carcinoma"[tiab] OR
```

"tongue cancer"[tiab] OR "tonsil cancer"[tiab] OR "tonsillar carcinoma"[tiab] OR

"sinonasal cancer"[tiab] OR "sinonasal carcinoma"[tiab] OR

"squamous cell carcinoma"[tiab])

("Positron-Emission Tomography"[MeSH Terms] OR

"Positron Emission Tomography Computed Tomography"[MeSH Terms] OR

"PET/CT"[tiab] OR "PET-CT"[tiab] OR "PETCT"[tiab] OR

"PET CT"[tiab] OR "FDG-PET"[tiab] OR "FDG PET"[tiab] OR

"18F-FDG"[tiab] OR "positron emission tomography"[tiab] OR

"SUV"[tiab] OR "standardized uptake value"[tiab])

("radiomics"[tiab] OR "radiomic"[tiab] OR "radiomics"[MeSH Terms] OR

"texture analysis"[tiab] OR "texture feature"[tiab] OR "texture features"[tiab] OR

"GLCM"[tiab] OR "GLRLM"[tiab] OR "GLSZM"[tiab] OR "NGTDM"[tiab] OR

"first-order features"[tiab] OR "shape features"[tiab] OR

"quantitative imaging"[tiab] OR "imaging biomarker"[tiab] OR

"handcrafted features"[tiab] OR "radiomic features"[tiab] OR

"deep learning"[tiab] OR "convolutional neural network"[tiab] OR "CNN"[tiab] OR

"transfer learning"[tiab] OR "deep features"[tiab] OR

"imaging phenotype"[tiab] OR "imaging signature"[tiab])

("prognosis"[MeSH Terms] OR "Diagnosis"[MeSH Terms] OR

"prediction"[tiab] OR "predictive"[tiab] OR "prognostic"[tiab] OR

"diagnostic"[tiab] OR "classification"[tiab] OR

"survival"[tiab] OR "overall survival"[tiab] OR "progression-free"[tiab] OR

"recurrence"[tiab] OR "treatment response"[tiab] OR

"AUC"[tiab] OR "C-index"[tiab] OR "concordance"[tiab] OR "ROC"[tiab] OR

"accuracy"[tiab] OR "sensitivity"[tiab] OR "specificity"[tiab] OR

"model"[tiab] OR "nomogram"[tiab])

MEDLINE (Ovid syntax) - 30 July 2026; retrieved see PubMed. Interface: Recorded for reproducibility; MEDLINE was searched through PubMed.

```
# MEDLINE (Ovid) Search Strategy

# PROSPERO: CRD420261319855

# Date: 2026-03-27



# 1  exp Head and Neck Neoplasms/

# 2  (head and neck cancer or head and neck cancers or HNSCC or HNC or oropharyngeal cancer or oropharyngeal carcinoma or laryngeal cancer or laryngeal carcinoma or hypopharyngeal cancer or hypopharyngeal carcinoma or nasopharyngeal cancer or nasopharyngeal carcinoma or NPC or oral cavity cancer or oral cancer or pharyngeal cancer or pharyngeal carcinoma or tongue cancer or tonsil cancer or tonsillar carcinoma or sinonasal cancer or squamous cell carcinoma).tw.

# 3  1 or 2

# 4  exp Positron-Emission Tomography/

# 5  exp Tomography, X-Ray Computed/

# 6  (PET/CT or PET-CT or PETCT or FDG-PET or FDG PET or 18F-FDG or positron emission tomography or computed tomography or SUV or standardized uptake value).tw.

# 7  4 or 5 or 6

# 8  (radiomics or radiomic or texture analysis or texture feature$ or GLCM or GLRLM or GLSZM or NGTDM or first-order features or shape features or quantitative imaging or imaging biomarker or deep learning or convolutional neural network or CNN or radiomic features or imaging signature or deep features or handcrafted features).tw.

# 9  3 and 7 and 8

# 10 limit 9 to english language
```

Cochrane CENTRAL - registered wave; retrieved 88. Interface: Cochrane Library Advanced Search.

```
# CENTRAL — Cochrane Central Register of Controlled Trials

# PROSPERO: CRD420261319855

# Date: 2026-03-27
```

```
#1 MeSH descriptor: [Head and Neck Neoplasms] explode all trees

#2 (head and neck cancer OR HNSCC OR HNC OR oropharyngeal OR laryngeal OR hypopharyngeal OR nasopharyngeal OR oral cavity cancer OR pharyngeal cancer OR squamous cell carcinoma):ti,ab,kw

#3 #1 OR #2

#4 MeSH descriptor: [Positron-Emission Tomography] explode all trees

#5 (PET/CT OR PET-CT OR FDG-PET OR FDG PET OR 18F-FDG OR positron emission tomography OR SUV):ti,ab,kw

#6 #4 OR #5

#7 (radiomics OR radiomic OR texture analysis OR texture feature* OR GLCM OR deep learning OR CNN OR quantitative imaging OR imaging biomarker OR radiomic features OR imaging signature):ti,ab,kw

#8 #3 AND #6 AND #7
```

Cochrane Library - registered wave; retrieved included in 88. Interface: Cochrane Library, all databases.

```
# PROSPERO: CRD420261319855

# Date: 2026-03-27

#1 (head and neck cancer OR HNSCC OR HNC OR oropharyngeal OR laryngeal OR hypopharyngeal OR nasopharyngeal OR oral cavity cancer OR pharyngeal cancer):ti,ab,kw

#2 (PET/CT OR PET-CT OR FDG-PET OR FDG PET OR positron emission tomography):ti,ab,kw

#3 (radiomics OR radiomic OR texture analysis OR texture feature* OR GLCM OR deep learning OR CNN OR quantitative imaging OR imaging biomarker OR radiomic features):ti,ab,kw

#4 #1 AND #2 AND #3
```

ASSIA - registered wave; retrieved 101. Interface: ProQuest, Saudi Digital Library.

```
# ASSIA – Applied Social Sciences Index & Abstracts

# PROSPERO: CRD420261319855

# Date: 2026-03-27

# S1: noft("head and neck cancer" OR "HNSCC" OR "oropharyngeal" OR "laryngeal cancer" OR "hypopharyngeal" OR "nasopharyngeal" OR "oral cavity cancer" OR "pharyngeal cancer" OR "squamous cell carcinoma")

# S2: noft("PET/CT" OR "PET-CT" OR "FDG-PET" OR "FDG PET" OR "positron emission tomography")
```

```
# S3: noft("radiomics" OR "radiomic" OR "texture analysis" OR "texture feature" OR "GLCM" OR "deep learning" OR "CNN" OR "quantitative imaging" OR "imaging biomarker" OR "radiomic features")

# S4: S1 AND S2 AND S3
```

Web of Science Core Collection - 30 July 2026, re-run 17 August 2026; retrieved 664. Interface: Advanced Search Query Builder; Editions: All; no date range, no language or document-type limit.

```
# Systematic Review: Radiomics Using Fused PET and CT Data in Head and Neck Cancer

# PROSPERO: CRD420261319855



#1  TS=(("head and neck" NEAR/3 (cancer* OR carcinom* OR neoplas* OR tumo$r*)) OR HNSCC OR HNC OR

        oropharyn* OR laryng* OR hypopharyn* OR nasopharyn* OR "oral cavity cancer" OR "oral cancer" OR

        pharyng* OR "tongue cancer" OR tonsil* OR sinonasal* OR "squamous cell carcinoma")

#2  TS=("PET/CT" OR "PET-CT" OR PETCT OR "PET CT" OR "FDG-PET" OR "FDG PET" OR "18F-FDG" OR

        "positron emission tomography" OR SUV OR "standardized uptake value" OR "standardised uptake value")

#3  TS=(radiomic* OR "texture analysis" OR "texture featur*" OR GLCM OR GLRLM OR GLSZM OR NGTDM OR

        "first-order featur*" OR "shape featur*" OR "quantitative imaging" OR "imaging biomarker*" OR

        "handcrafted featur*" OR "deep learning" OR "convolutional neural network*" OR CNN OR

        "transfer learning" OR "deep featur*" OR "imaging phenotype*" OR "imaging signature*")

#4  #1 AND #2 AND #3

TS=(("head and neck" NEAR/3 (cancer* OR carcinom* OR neoplas* OR tumo$r*)) OR HNSCC OR HNC OR oropharyn* OR laryng* OR hypopharyn* OR nasopharyn* OR "oral cavity cancer" OR "oral cancer" OR pharyng* OR "tongue cancer" OR tonsil* OR sinonasal* OR "squamous cell carcinoma") AND TS=("PET/CT" OR "PET-CT" OR PETCT OR "PET CT" OR "FDG-PET" OR "FDG PET" OR "18F-FDG" OR "positron emission tomography" OR SUV OR "standardized uptake value" OR "standardised uptake value") AND TS=(radiomic* OR "texture analysis" OR "texture featur*" OR GLCM OR GLRLM OR GLSZM OR NGTDM OR "first-order featur*" OR "shape featur*" OR "quantitative imaging" OR "imaging biomarker*" OR "handcrafted featur*" OR "deep learning" OR "convolutional neural network*" OR CNN OR "transfer learning" OR "deep featur*" OR "imaging phenotype*" OR "imaging signature*")
```

IEEE Xplore - 17 August 2026; retrieved 80. The query was three blocks joined by AND, translated from the strategy of record into IEEE command syntax within the platform's limit of 25 terms per clause: 14 population terms, 10 modality terms, 17 method terms. The exact string as pasted was not saved to the project record; it is therefore not reproduced here, and it has not been reconstructed. The 80 retrieved records, their DOIs and the full screening decisions are in the review data, so the yield is auditable even though the string is not.

OpenAlex - 17 August 2026; retrieved largest single contributor to the update wave. Executed programmatically, not pasted into a search box; the query is embodied in the script filed with the review data.

Crossref - 31 July 2026; proceedings 17 August 2026; retrieved 2,400 swept; 65 chapters. Executed programmatically, not pasted into a search box; the queries are embodied in the scripts filed with the review data.

Embase - attempted 17 August 2026. Entitlement absent. The platform returned: "You are successfully signed in to Elsevier. Your account doesn't have direct access to Embase, but you might have access to Embase via your institution." No search interface was reachable, so no search was run and none is claimed.

Scopus - attempted 17 August 2026. Entitlement absent. The advanced search form does not load for an unentitled account; the request redirects to Scopus Preview, which exposes source and author records only and cannot run a document search.
